# Transcript analyses reveal a comprehensive role of abscisic acid in modulating fruit ripening in Chinese jujube

**DOI:** 10.1186/s12870-019-1802-2

**Published:** 2019-05-08

**Authors:** Zhong Zhang, Chenxuan Kang, Shuyi Zhang, Xingang Li

**Affiliations:** 1 College of Forestry, Northwest A&F University, Yangling, 712100 Shaanxi China; 2 Key Comprehensive Laboratory of Forestry of Shaanxi Province, Northwest A&F University, Yangling, 712100 Shaanxi China; 3 Research Centre for Jujube Engineering and Technology of State Forestry and Grassland Administration, Northwest A&F University, Yangling, 712100 Shaanxi China; 4 Forestry Administration of Linwei District, Weinan, 714000 Shaanxi China

**Keywords:** Chinese jujube (*Ziziphus jujuba*), Abscisic acid, Fruit ripening, Non-climacteric, Transcriptome sequencing

## Abstract

**Background:**

Chinese jujube (*Ziziphus jujuba* Mill.) is a non-climacteric fruit; however, the underlying mechanism of ripening and the role of abscisic acid involved in this process are not yet understood for this species.

**Results:**

In the present study, a positive correlation between dynamic changes in endogenous ABA and the onset of jujube ripening was determined. Transcript analyses suggested that the expression balance among genes encoding *nine-cis-epoxycarotenoid dioxygenase* (*ZjNCED3*), *ABA-8′-hydroxylase* (*ZjCYP707A2*), and *beta-glucosidase* (*ZjBG4*, *ZjBG5*, *ZjBG8*, and *ZjBG9*) has an important role in maintaining ABA accumulation, while the expression of a receptor (*ZjPYL8*), *protein phosphatase 2C* (*ZjPP2C4–8*), and *sucrose nonfermenting 1-related protein kinase 2* (*ZjSnRK2–2* and *ZjSnRK2–5*) is important in regulating fruit sensitivity to ABA applications. In addition, white mature ‘Dongzao’ fruit were harvested and treated with 50 mg L^− 1^ ABA or 50 mg L^− 1^ nordihydroguaiaretic acid (NDGA) to explore the role of ABA in jujube fruit ripening. By comparative transcriptome analyses, 1103 and 505 genes were differentially expressed in response to ABA and NDGA applications on the 1st day after treatment, respectively. These DEGs were associated with photosynthesis, secondary, lipid, cell wall, and starch and sugar metabolic processes, suggesting the involvement of ABA in modulating jujube fruit ripening. Moreover, ABA also exhibited crosstalk with other phytohormones and transcription factors, indicating a regulatory network for jujube fruit ripening.

**Conclusions:**

Our study further elucidated ABA-associated metabolic and regulatory processes. These findings are helpful for improving strategies for jujube fruit storage and for gaining insights into understand complex non-climacteric fruit ripening processes.

**Electronic supplementary material:**

The online version of this article (10.1186/s12870-019-1802-2) contains supplementary material, which is available to authorized users.

## Background

Chinese jujube (*Ziziphus jujuba* Mill.) is a popular fruit crop species that is native to China and is highly desired by consumers worldwide due to the abundant nutritional and health benefits of the fruit [1, 2]. However, the flesh jujube fruit has a very short shelf-life underlined by rapid dehydration or water-soaking deterioration within 2–3 days after harvest [3]. Therefore, fruit storage and quality maintenance have been among the most urgent challenges in the development of the jujube industry, whereas knowledge related to its ripening characterization and regulation is lacking. Over the past few decades, great strides have been made in elucidating the regulation of fruit ripening [4]; in particular, ethylene and abscisic acid (ABA) are recognized as the most important phytohormones that are directly or indirectly involved in the ripening of both climacteric and non-climacteric fruit [5, 6]. Recently, Chinese jujube has been characterized as a non-climacteric fruit, while a basal level of ethylene is still needed to maintain full fruit maturity [7]. These findings also reveal that the regulation of ripening is relatively complex and that there is a further need to explore these mechanisms to deepen our understanding of the ripening of Chinese jujube fruit.

With regard to ABA, the presence of dramatically increased levels in fruit during the onset of ripening has been reported in several fruit crop species, including grape [8], sweet cherry [9], cucumber [10], watermelon [11], and persimmon [5], which points to a role for ABA in triggering the onset of fruit ripening [8]. Moreover, applications of exogenous ABA and nordihydroguaiaretic acid (NDGA, an inhibitor of ABA biosynthesis) have enabled us to identify ABA-dependent pathways [12, 13]; increased numbers of research findings have suggested a positive role for ABA in promoting the metabolism and accumulation of soluble sugars [12, 14], formation of peel color [15, 16], and modification of cell wall catabolism [17], thereby accelerating ripening processes [5]. Fruit ripening is a highly integrated process that involves hormone control and crosstalk, as well as alterations to the numbers of transcripts of transcription factors (TFs) [18, 19]. Increasing amounts of transcriptome sequencing data have provided us with more extensive insight into molecular mechanisms and regulatory networks. For example, ABA regulates the expression of the vast majority of genes involved in strawberry fruit ripening [19], and ABA also has a potential influence on the metabolism and signaling of ethylene, auxin, and gibberellins (GAs) [12, 20–22]. Nevertheless, the involvement of ABA in regulating fruit ripening has scarcely been reported in the non-climacteric fruit of Chinese jujube.

**Fig. 1:**
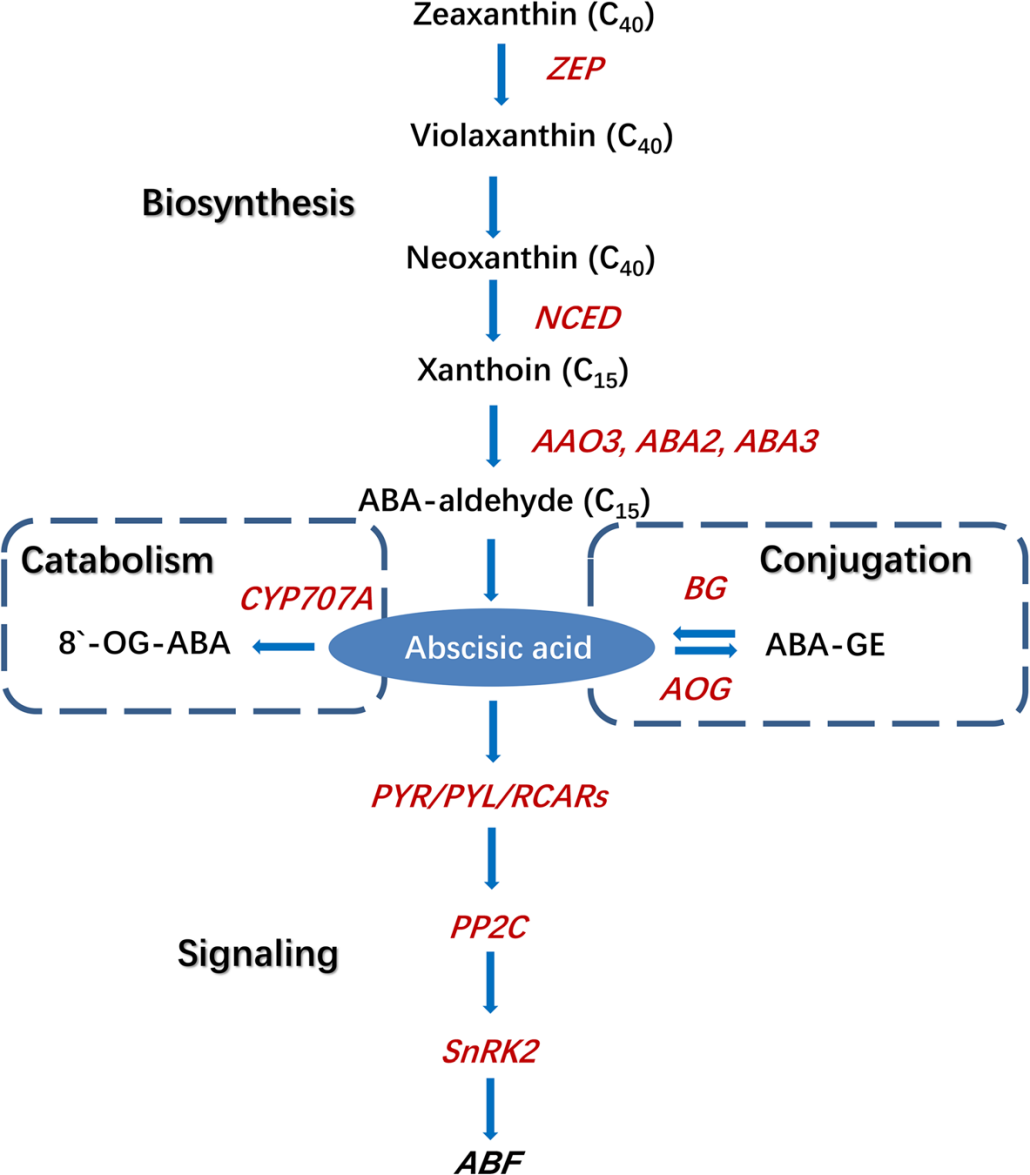
ABA biosynthesis, catabolism, conjugation, and signaling pathway according to Leng et al. (2014) [5]. The expression of genes colored in red were determined in the manuscript

In the present study, we aimed to explore the putative role of ABA in the regulation of jujube fruit ripening. The dynamic changes in ABA levels during fruit ripening processes were determined, and fruit undergoing the onset of ripening were harvested and treated with exogenous ABA and NDGA to investigate the ABA-dependent pathways and metabolic processes. Using the jujube reference genome (LPXJ00000000.1), we identified genes involved in ABA biosynthesis, metabolism, and signaling (Fig. 1), and their expression during fruit development and ripening was determined by qRT-PCR. In addition, we simultaneously examined ABA-associated ripening metabolism and regulatory networks comprising hormone crosstalk and transcription factor activity via transcriptome sequencing. These results provide insights into the ABA network in non-climacteric ripening and into further improvement strategies for jujube fruit storage.

## Results

### Accumulation of endogenous ABA along with Chinese jujube fruit development and ripening

**Fig. 2:**
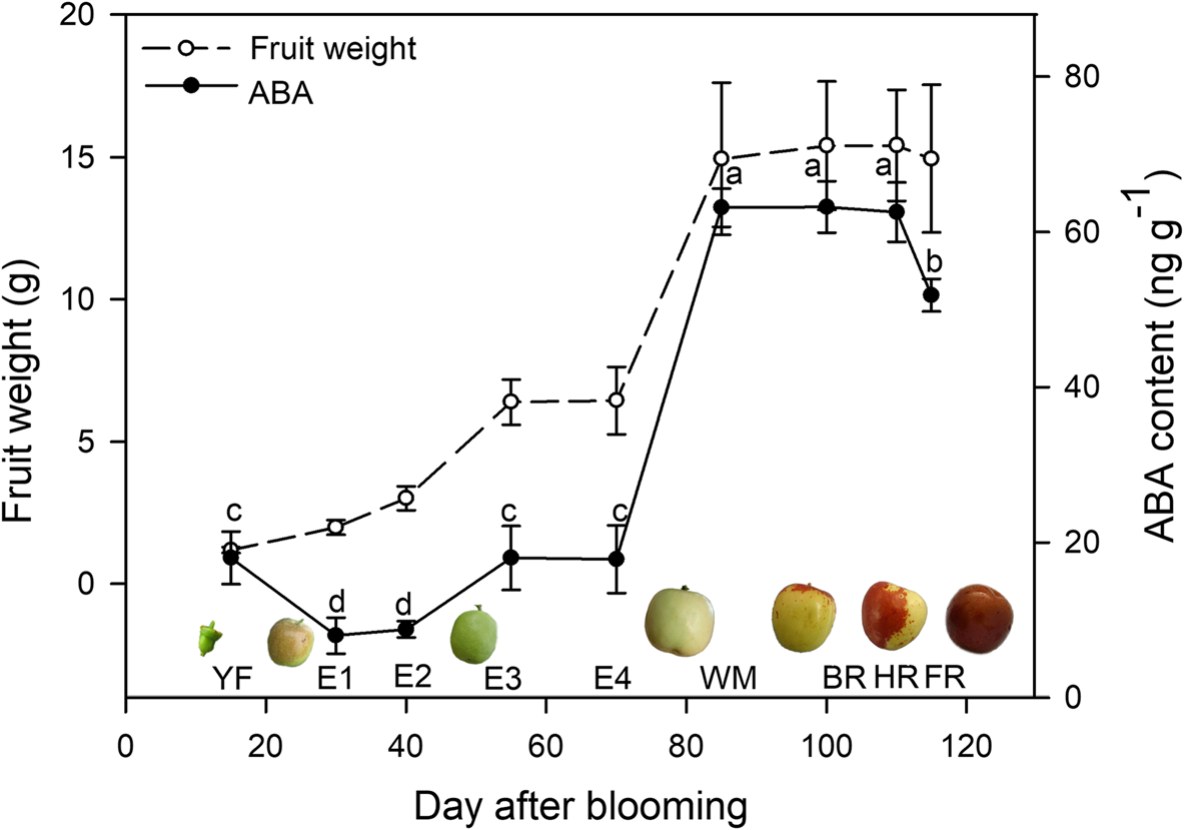
Dynamic changes in fruit weight and endogenous ABA content during the ‘Dongzao’ fruit development and ripening process. YF, young fruit; E, enlarging fruit; WM, white mature; BR, beginning red; HR, half red; FR, full red. The different letters over the bars represent significant differences between the mean values (*p* < 0.05, Duncan test)

The whole dynamic growth of ‘Dongzao’ fruit conformed to a double-sigmoidal pattern (Fig. 2), with two rapid growth phases from E2 to E3, followed by E4 to WM. The WM stage was defined as the onset of fruit ripening, during which the fruit color turned whitish-green and the weight accumulation rate clearly decreased. The content of endogenous ABA was determined along with fruit growth processes, with low and stable levels in immature fruit; the content displayed a maximum increase at WM and remained at a high level in BR and HR fruit. Afterwards, the content decreased at the FR stage with a relatively lower level than that observed in the HR fruit. We performed a statistical analysis by using a Person correlation model, and the results revealed a significant correlation between the ABA content and fruit weight, with an R squared value = 0.9254 (*p* < 0.0001, two tailed).

### Relative expression of ABA biosynthesis genes during Chinese jujube fruit development and ripening

**Fig. 3:**
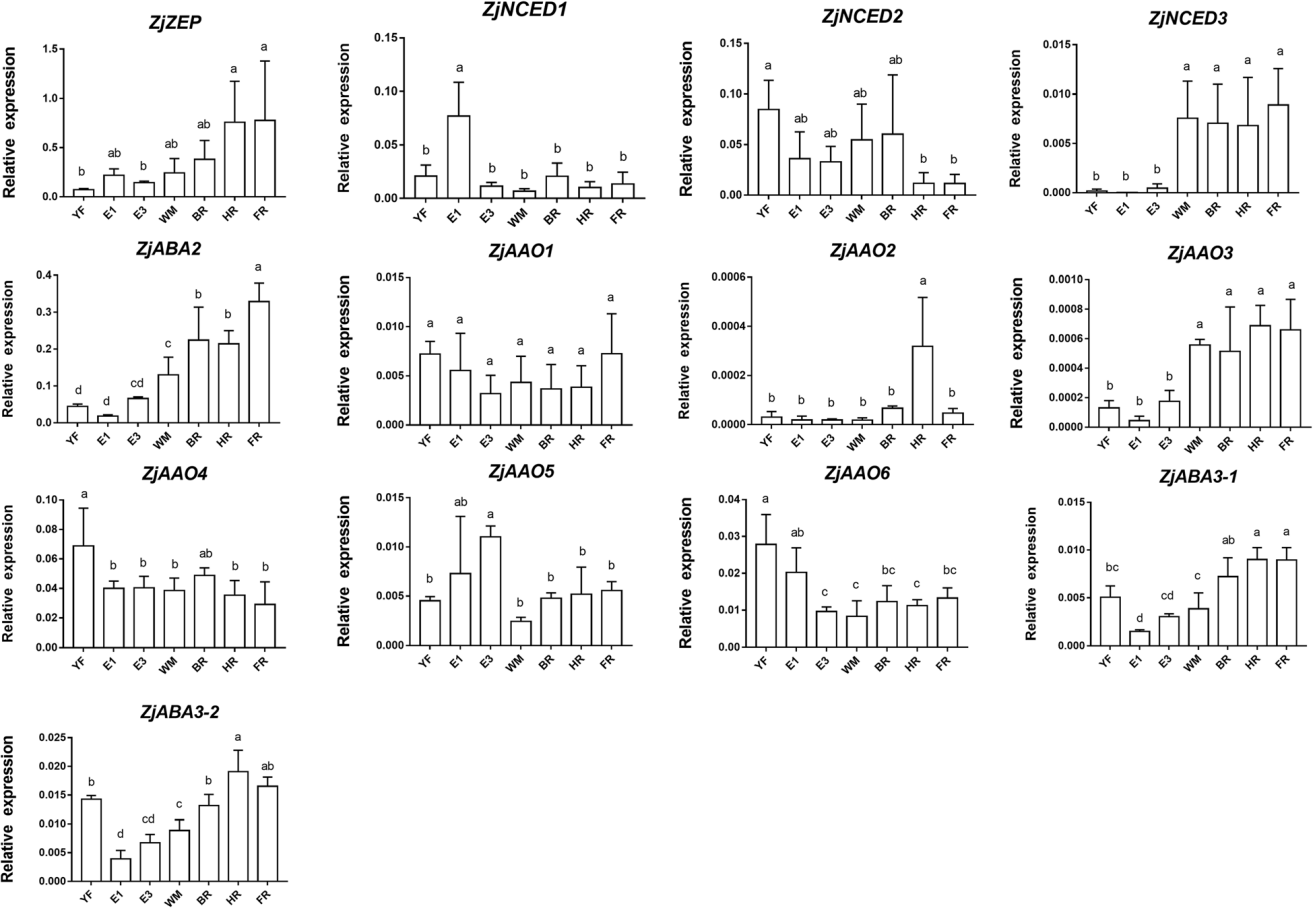
Relative expression of ABA biosynthesis genes during ‘Dongzao’ fruit development and ripening. YF, young fruit; E, enlarging fruit; WM, white mature; BR, beginning red; HR, half red; FR, full red. The different letters over bars represent significant differences between the mean values (*p* < 0.05, Duncan test)

In the ABA biosynthesis pathway, 13 genes were identified from the reference jujube genome, including 1 *zeaxanthin epoxidase* (*ZEP*), 3 *NCED*, 1 *xanthoxin dehydrogenase* (*ABA2*), 6 *abscisic aldehyde oxidase* (*AAO*), and 2 *molybdenum cofactor sulfurtransferase* (*ABA3*) genes. The relative expression of *ZjZEP* did not significantly change during jujube fruit development and ripening (Fig. 3). *ZjNCED1* was consistently expressed during the whole growth process, except at the E1 stage, during which relatively high expression was detected. *ZjNCED2* also showed a stable expression pattern, but the level was slightly lower at the HR and FR stages than at the other stages. *ZjNCED3* showed rapid increase in expression at the WM stage and maintained a relatively high level during fruit ripening. *ZjABA2* was not highly expressed in immature fruit, but the number of transcripts increased significantly after the WM stage. *ZjAAO3* was expressed at a low level, but its expression rapidly increased at WM and remained high during ripening, while the relative expression of *ZjAAO1*, *ZjAAO2*, *ZjAAO4*, *ZjAAO5*, and *ZjAAO6* did not correlate with ABA accumulation. In addition, *ZjABA3–1* and *ZjABA3–2* showed a similar trend in which their expression level was higher after the WM stage than in the immature fruit.

### Relative expression of ABA metabolism genes during Chinese jujube fruit development and ripening

**Fig. 4:**
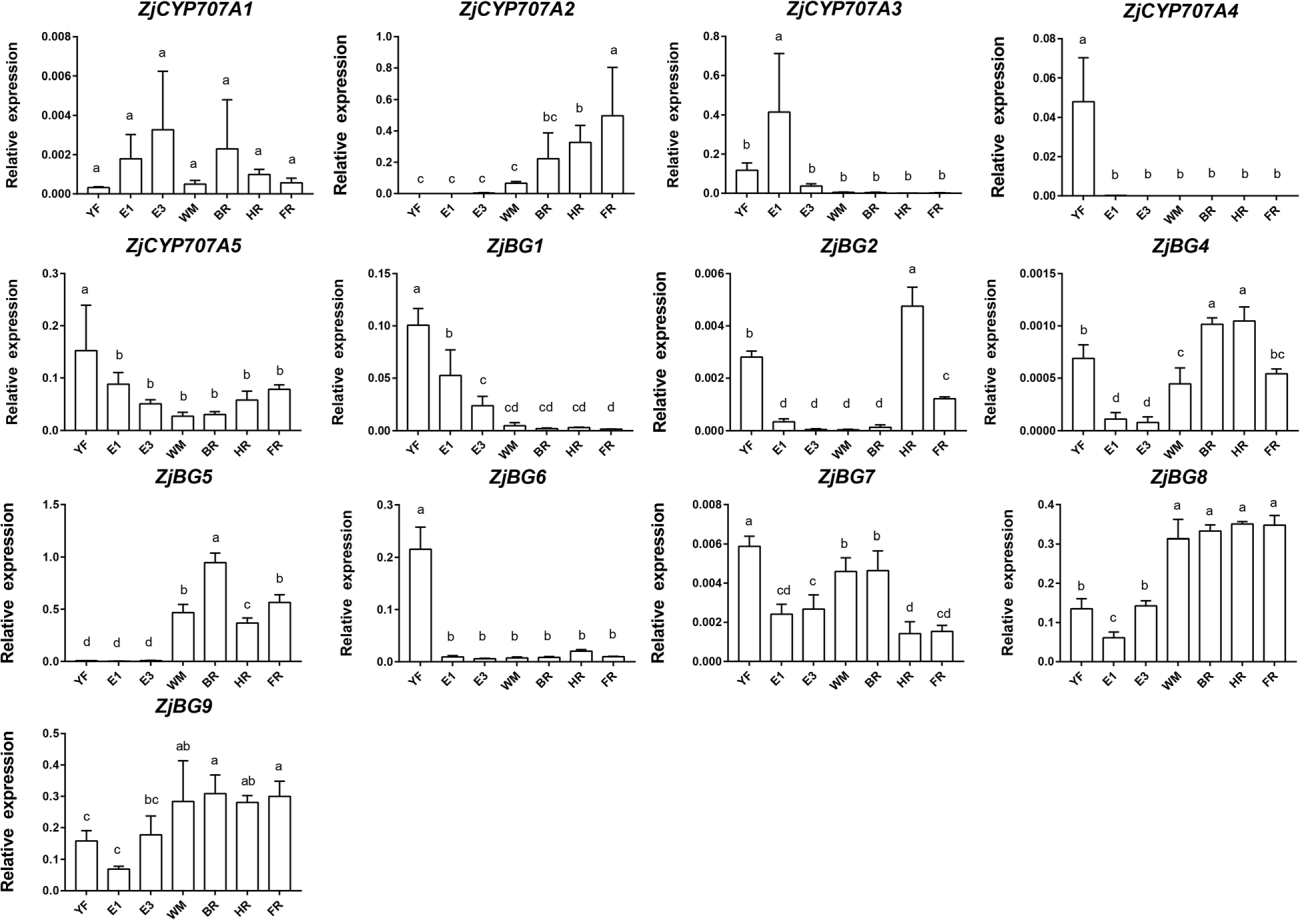
Relative expression of ABA metabolism genes during ‘Dongzao’ fruit development and ripening. YF, young fruit; E, enlarging fruit; WM, white mature; BR, beginning red; HR, half red; FR, full red. The different letters over the bars represent significant differences between the mean values (*p* < 0.05, Duncan test)

In the ABA catabolism pathway, we identified five *CYP707A*s. The relative expression of *ZjCYP707A1* was low and did not significantly change during fruit development and ripening (Fig. 4). *ZjCYP707A2* exhibited relatively low expression in immature fruit; however, its transcription strongly increased, which was accompanied by ABA accumulation, and reached a maximum level at the FR stage. *ZjCYP707A3* showed relatively high expression in E1 fruit, but this expression decreased to a very low level afterwards. *ZjCYP707A4* was highly expressed only at the YF stage. *ZjCYP707A5* had a relatively high expression at the YF stage, but the expression decreased afterward, with no clear difference.

In the ABA homeostasis pathway, 8 *BG*s were identified. Among them, *ZjBG4*, *ZjBG5*, *ZjBG8*, and *ZjBG9* had similar expression patterns along with ABA accumulation; the expression levels of these genes were low in immature fruit but then increased after the WM stage. *ZjBG1* showed higher expression in the YF and E1 stages than in the other stages, but the expression then decreased and did not significantly change. *ZjBG2* showed higher expression at the HR, FR and YF stages than at the other stages. *ZjBG6* showed relatively high expression at YF and did not significantly change in other stages. *ZjBG7* showed higher expression in the YF, WM, and BR stages than in the other stages. In addition, we also identified two *ABA-glucosyltransferases* (*AOG*), but their expression levels were extremely low and were not shown in the figure.

### Relative expression of ABA signaling pathway genes during Chinese jujube fruit development and ripening

**Fig. 5:**
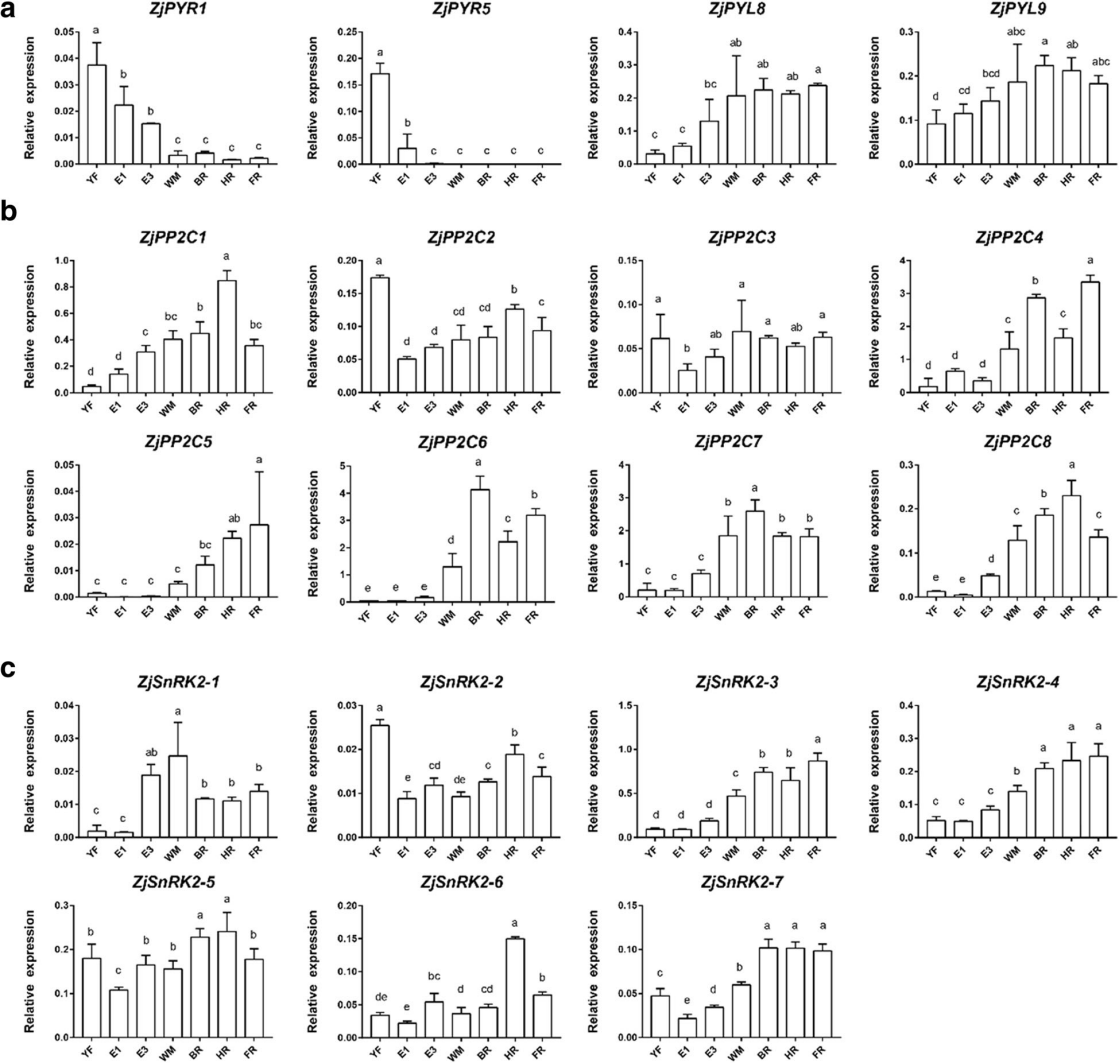
Relative expression of ABA signaling pathway genes during ‘Dongzao’ fruit development and ripening. **a**. ABA receptors. **b**. *PP2C* genes. **c**. *SnRK2* genes. *YF* young fruit, *E* enlarging fruit, *WM* white mature, *BR* beginning red, *HR* half redm, *FR* full red. The different letters over the bars represent significant differences between the mean values (*p* < 0.05, Duncan test)

With respect to the signaling pathway, 6 genes encoding ABA receptors as well as *PYR/PYL/RCAR* were identified. *ZjPYR1* and *ZjPYR5* showed relatively high expression in the YF and E1 stages, but their expressions were very low after the onset of ripening (Fig. 5a). The expression levels of *ZjPYL8* and *ZjPYL9* were low in immature fruit but slightly increased after WM. In addition, *ZjPYL2* and *ZjPYL4* were not expressed in jujube fruit.

Among the eight identified *PP2C*s, *ZjPP2C4*, *ZjPP2C5*, *ZjPP2C6*, *ZjPP2C7*, and *ZjPP2C8* had similar expression patterns along with ABA accumulation, with rapidly increased expression at the WM stage (Fig. 5b). However, *ZjPP2C1* showed lower expression in the YF and E1 stages but higher expression in HR fruit. *ZjPP2C2* exhibited relatively high expression in the YF stage, but its expression was consistent afterwards. The relative expression of *ZjPP2C3* also did not significantly change during the whole fruit growth process.

Seven *SnRK2*s were identified from the Chinese jujube genome. *ZjSnRK2–3*, *ZjSnRK2–4* and *ZjSnRK2–7* showed relatively low expression in immature fruit, but their expression significantly increased with ABA accumulation. In addition, *ZjSnRK2–1* showed lower expression in the YF and E1 stages than in the other stages, but its expression increased beginning at the E3 stage, with maximum expression occurring at the WM stage (Fig. 5c). *ZjSnRK2–2* had relatively high expression at YF, but the expression remained consistently in the other stages. *ZjSnRK2–5* and *ZjSnRK2–6* showed relatively consistent expression patterns during fruit development and ripening, although *ZjSnRK2–6* showed maximum expression in HR fruit.

### Changes in maturity and hormone levels of WM Chinese jujube fruit in response to ABA and NDGA

**Fig. 6:**
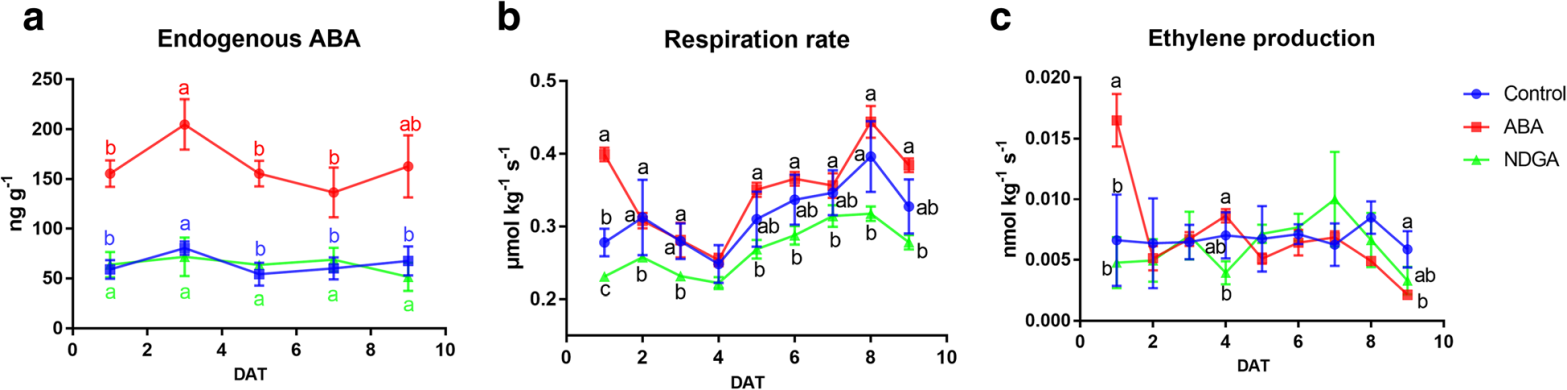
Changes in hormone levels, respiration rate, and ethylene production of WM ‘Dongzao’ fruit in response to exogenous ABA and NDGA treatment. **a**. Endogenous ABA content. **b**. Respiration rate. **c**. Ethylene production. The different letters over the bars represent significant differences between the mean values (*p* < 0.05, Duncan test)

To explore the role of ABA in regulating Chinese jujube fruit ripening, WM fruit were harvested and treated with exogenous ABA or NDGA. As expected, the endogenous ABA concentration strongly increased and remained at a high level during shelf storage in ABA-treated fruit (Fig. 6a). At 3 DAT, a moderate peak in ABA content was found in both CK- and ABA-treated fruit, although the content was slightly lower in NDGA-treated fruit at this time point. The changes in respiration rate first tended to decrease but then tended to moderately increase during storage (Fig. 6b). Exogenous ABA treatment highly increased the respiration rate at 1 DAT, but the effect was subsequently not as significant. NDGA significantly inhibited fruit respiration, as the level decreased in the treated fruit compared with the control fruit. In addition, ethylene production was very low, and ABA treatment induced a significant increase at 1 DAT, while NDGA had a limited effect on ethylene production (Fig. 6c).

### Identification of DEGs from comparative transcriptome analysis

**Fig. 7:**
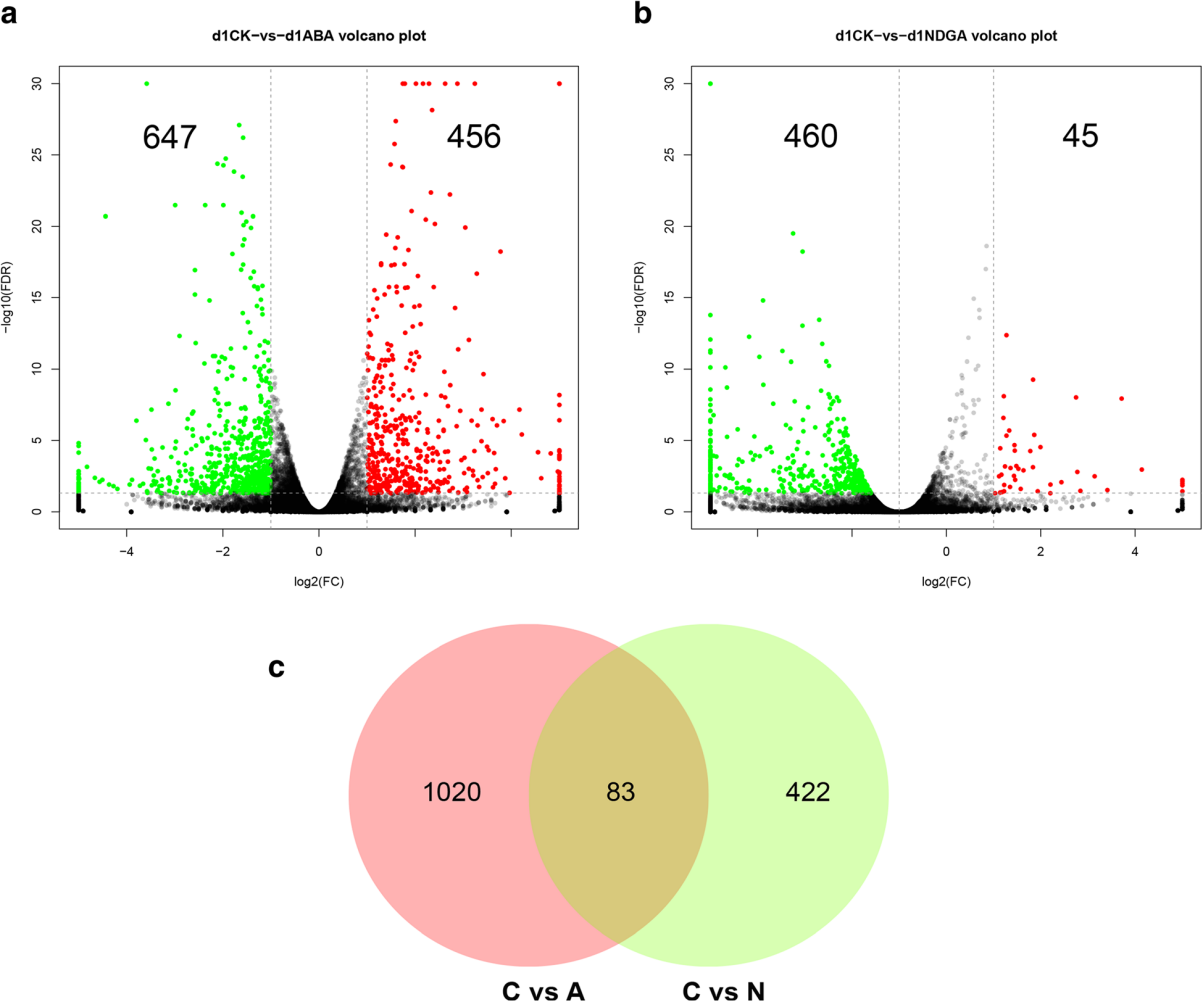
Identification of DEGs in response to ABA and NDGA treatment at 1 DAT. **a**. Volcano plot analysis between the control (C) and ABA (A)-treated fruit. The green plot indicates the downregulated transcripts, and the red plot represents the upregulated transcripts. The threshold for DEGs was set at a fold change ≥2 and an FDR ≤ 0.05. **b**. Volcano plot analysis between the control (C) and NDGA (N)-treated fruit. **c**. Venn diagram showing the number of specific and common DEGs between the ABA and NDGA treatments

To investigate the molecular basis of ABA-associated ripening processes, the WM jujube fruit that underwent ABA and NDGA treatment were subjected to transcriptome sequencing at 1 DAT. In total, 41.26 Gb of raw sequencing data comprising 275,139,538 reads were generated; after quality control, 271,970,678 clean reads were used for further analysis (Additional file 1). By alignment with the jujube genome, an average of 87.42% reads were uniquely mapped for each library (Additional file 2), and the relative expression levels of 31,031 transcripts were calculated. By comparative transcriptome analysis, 1103 DEGs were identified, with 456 upregulated and 647 downregulated transcripts detected in response to exogenous ABA (Fig. 7a). Moreover, 505 gene transcripts significantly changed in response to NDGA treatment, of which 45 genes were upregulated and 460 were downregulated (Fig. 7b). When these outputs were compared, 83 DEGs were simultaneously induced by both ABA and NDGA (Fig. 7c).

### Functional enrichment of DEGs by GO, KEGG, and MapMan analyses

**Fig. 8:**
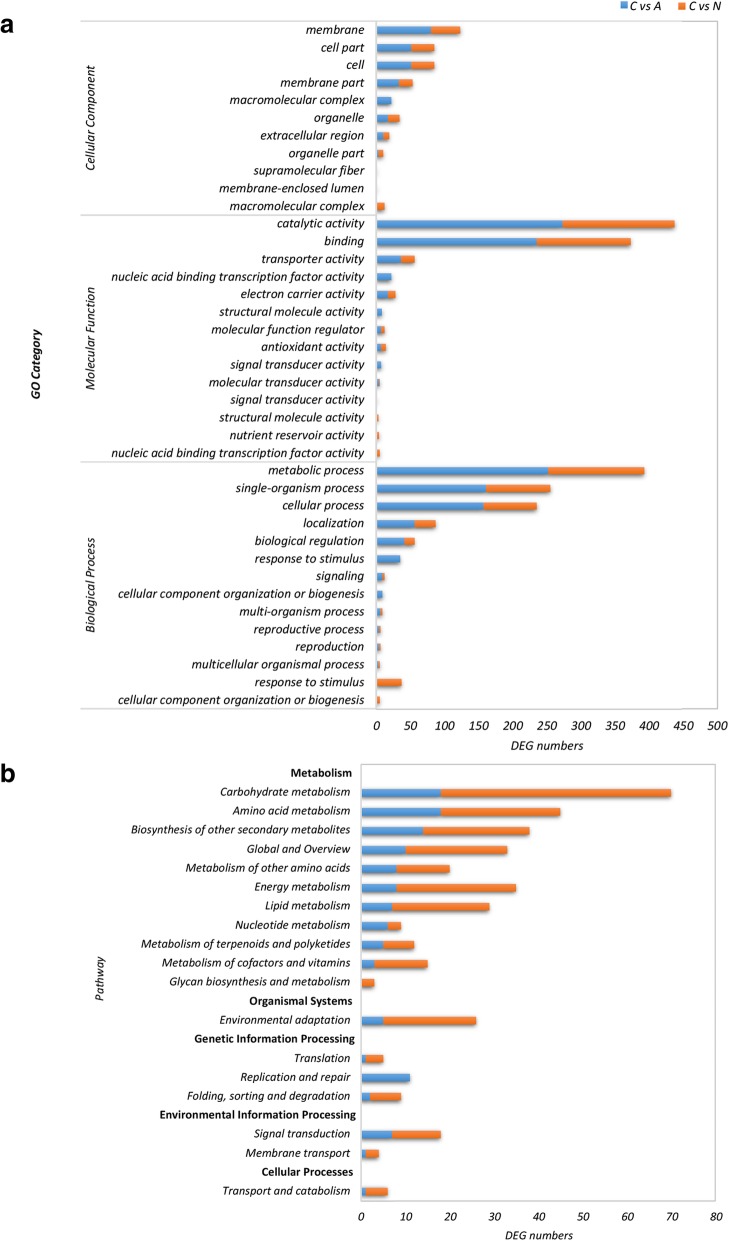
Functional enrichment of DEGs induced by ABA and NDGA treatments. **a**. GO enrichment analysis. **b**. KEGG pathway analysis

To reveal the differences in ABA and NDGA treatment effects on jujube fruit ripening, we enriched the putative function of specific DEGs using GO annotation, generating 31 and 32 classes at level 2 for the ABA (1020) and NDGA (422) treatments, respectively (Additional file 3). The membrane, cell part, and cell classes were highly represented in the cellular component (Fig. 8a). The catalytic activity and binding classes were highly enriched in molecular function. Last, the metabolic process, single-organism process, and cellular process were involved in the most highly enriched biological processes.

The KEGG pathway analysis enriched the DEGs into 83 and 72 pathways for the ABA and NDGA treatments, respectively (Additional file 4). These pathways were mostly associated with 10 metabolic processes (Fig. 8b), including those affecting carbohydrates, amino acids, the biosynthesis of other secondary metabolites, other amino acids, energy, lipids, nucleotides, terpenoids and polyketides, cofactors and vitamins, and glycan biosynthesis and metabolism. Moreover, genes related to environmental adaptation, translation, replication and repair, folding, sorting and degradation, signaling transduction, membrane transport, and transport and catabolism were also highly represented in pathway enrichment in response to the treatments.

**Fig. 9:**
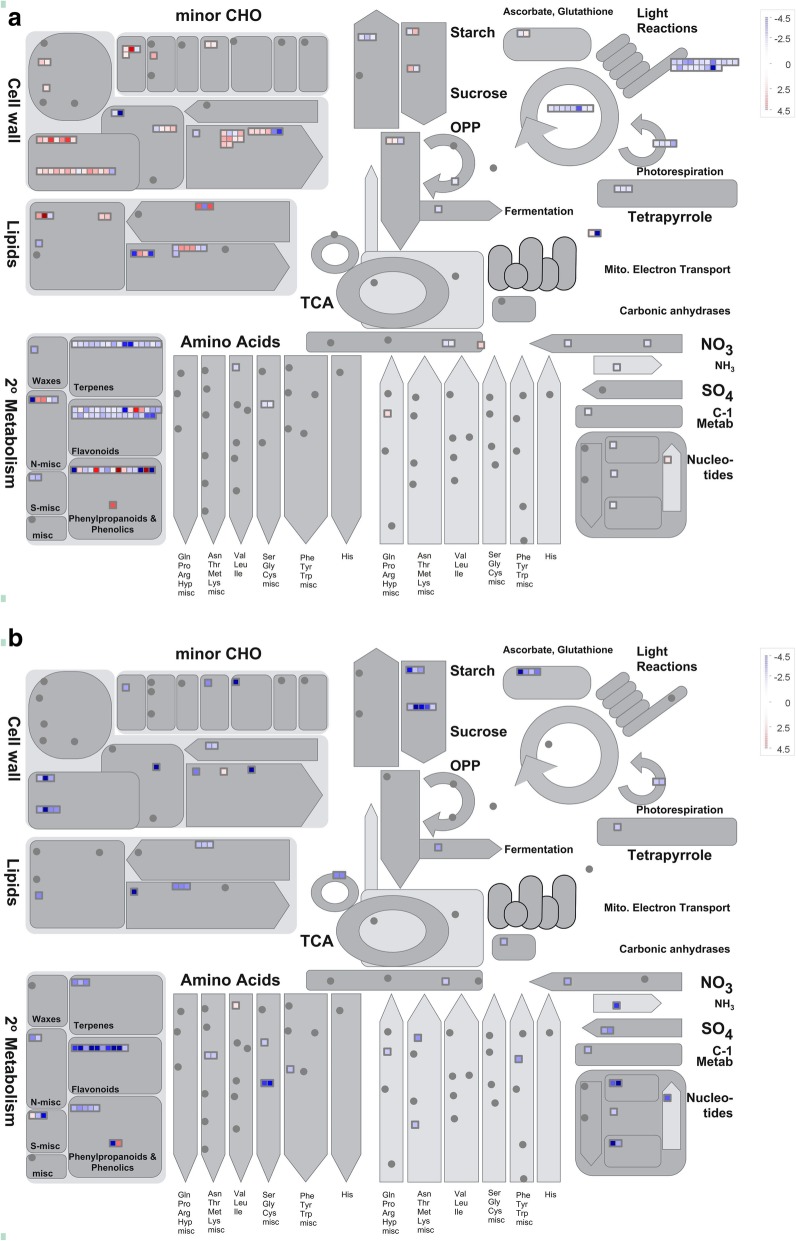
Overview of DEGs involved in metabolic processes according to MapMan ontology. **a**. DEGs induced by ABA treatment. **b**. DEGs induced by NDGA treatment. The blue squares indicate downregulation, and the red squares represent upregulation. The scale bar is shown as the log_2_ (fold change) value

In addition, MapMan analysis allowed us to gain an overview of the transcriptional changes in metabolic processes upon ABA and NDGA treatments. In response to exogenous ABA, DEGs related to photosynthesis metabolism and several secondary metabolite (terpene and flavonoid) processes were significantly suppressed, while cell wall modification, lipid metabolism, and starch and sucrose metabolism were somewhat promoted (Fig. 9a, Additional file 5). In addition, NDGA significantly inhibited these metabolic processes, which included secondary metabolism, cell wall modification, lipid metabolism, amino acid metabolism, and starch and sucrose metabolism (Fig. 9b, Additional file 5).

### DEGs related to plant hormone metabolism and signaling in response to ABA and NDGA treatments

**Fig. 10:**
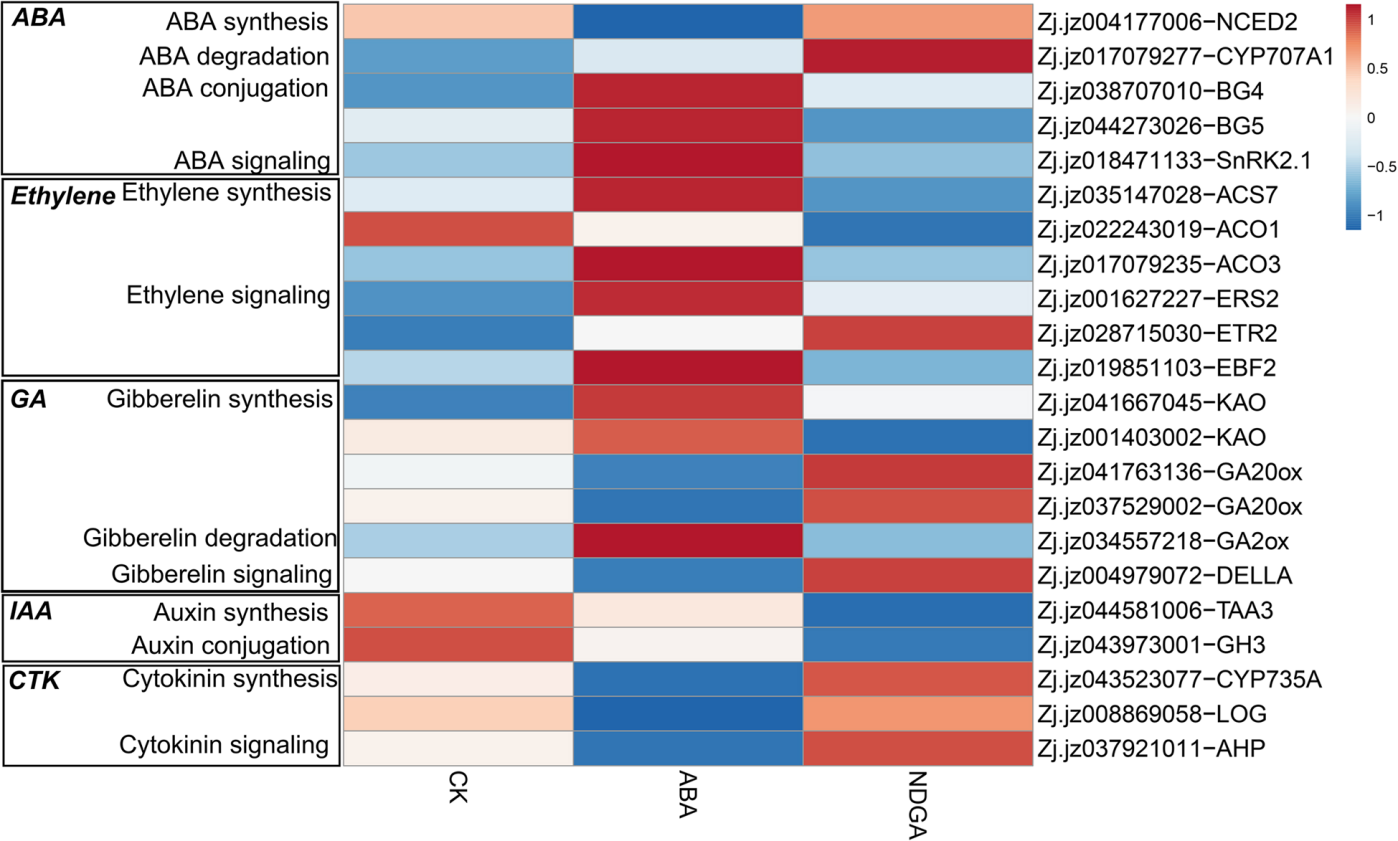
Heatmap of DEGs related to plant hormone metabolic and signaling pathways in response to ABA and NDGA treatments. The blue squares indicate downregulation, and the red squares represent upregulation. The scale bar is shown as the log_2_ (fold change) value

A number of DEGs related to phytohormone metabolism and signaling were identified (Fig. 10, Additional file 6). In ABA metabolism, exogenous ABA downregulated the expression of *ZjNCED2* (Zj.jz004177006) and upregulated the expression of two beta-glucosidases (*ZjBG4*, Zj.jz038707010; *ZjBG5*, Zj.jz044273026), while NDGA promoted the transcription of *ZjCYP707A1* (Zj.jz017079277) and suppressed the transcription of *ZjBG5* (Zj.jz044273026). In the signaling pathway, gene expression was not significantly induced by exogenous ABA or NDGA.

Transcripts of genes controlling ethylene biosynthesis, including 1-aminocyclopropane-1-carboxylate synthase (*ACS7*, Zj.jz035147028) and 1-aminocyclopropane-1-carboxylate oxidase (*ACO3*, Zj.jz017079235), significantly accumulated in response to ABA treatment. However, in response to NDGA, the expression of *ACO1* (Zj.jz022243019) was downregulated. In ethylene signaling, expression of the receptor *ERS2* (Zj.jz001627227) was strengthened by ABA, and *ETR2* (Zj.jz028715030) was highly upregulated in response to NDGA. The other genes involved in signaling were barely induced by either treatment; in addition, an ethylene-insensitive 3-binding F-box (*EBF*, Zj.jz019851103), which modulates ethylene-insensitive 3-like (*EIN3/EIL*) proteins at the posttranslational level, was upregulated by ABA but not significantly by NDGA.

The expression of an *ent*-kaurenoic acid hydroxylase (*KAO*, Zj.jz041667045) involved in the early steps of GA biosynthesis was upregulated in response to ABA, and the expression of *KAO* (Zj.jz001403002) was downregulated by NDGA. Moreover, two GA-20-oxidases (*GA20ox*, Zj.jz041763136, Zj.jz037529002), which participate in the biosynthesis of active GA, were downregulated in response to exogenous ABA. For GA degradation, a GA-2-oxidase (*GA2ox*, Zj.jz034557218) displayed relatively high expression in ABA-treated fruit. With respect to the signaling pathway, the transcription of *DELLA1* (Zj.jz004979072) was downregulated by ABA but was not significantly induced by NDGA.

The relative expression of genes involved in auxin metabolism and signaling was barely induced by exogenous ABA; however, a tryptophan aminotransferase (*TAA*, Zj.jz044581006) gene involved in the tryptophan-dependent pathway for auxin biosynthesis and a gretchen hagen 3 (*GH3*, Zj.jz043973001) gene involved in conjugation were simultaneously downregulated by NDGA treatment. In addition, exogenous ABA downregulated the expression of two genes controlling cytokinin biosynthesis, including *CYP735A* (Zj.jz043523077), which encodes a cytokinin trans-hydroxylase, and *LOG* (Zj.jz008869058), which encodes a cytokinin riboside 5′-monophosphate phosphoribohydrolase. In signaling, only a histidine-containing phosphotransferase (*AHP*) was downregulated by ABA, and NDGA showed a limited effect on the expression levels of these genes.

### DEGs related to TFs in response to ABA and NDGA treatments

**Fig. 11:**
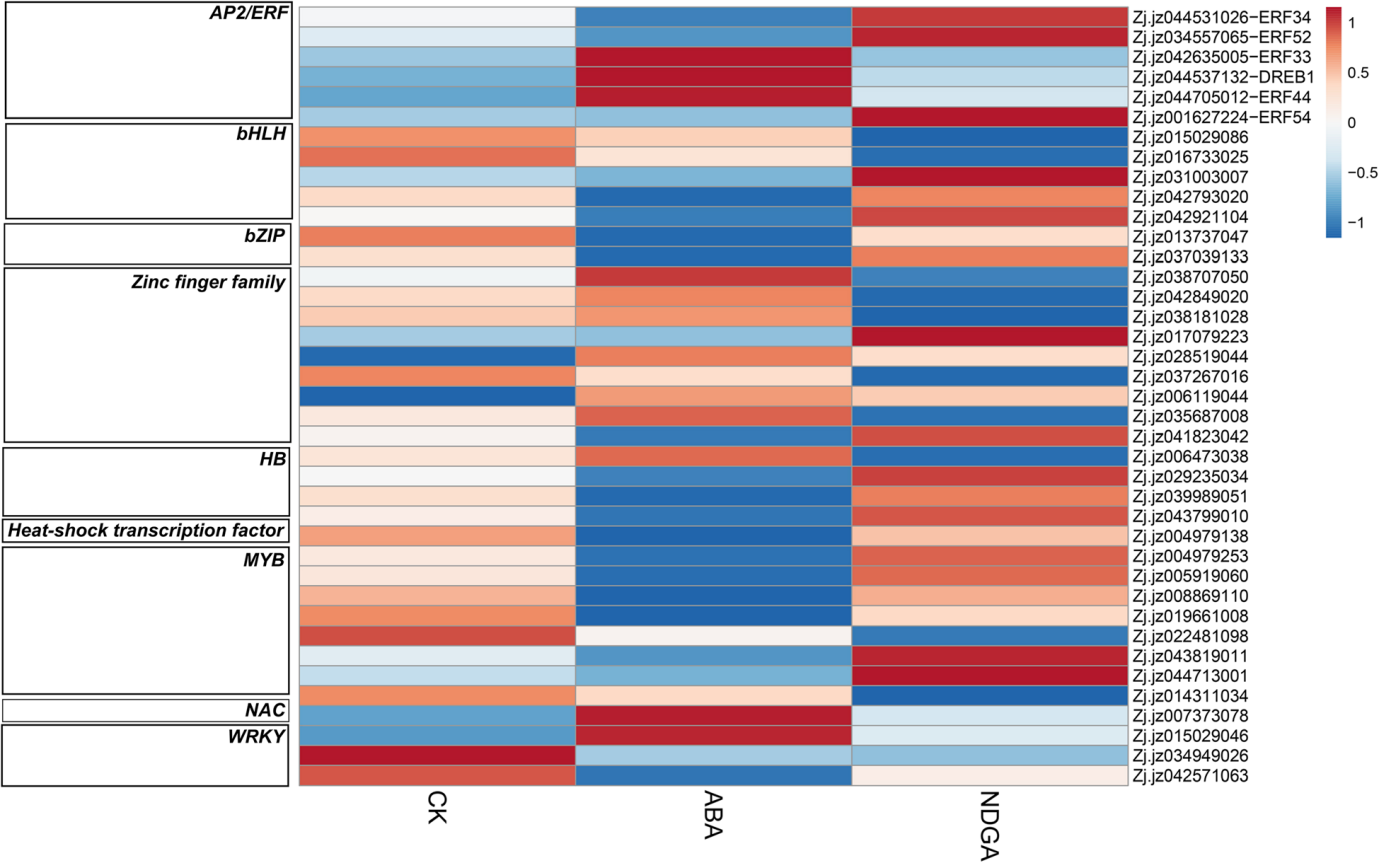
Heatmap of DEGs related to TFs in response to ABA and NDGA treatments. The blue squares indicate downregulation, and the red squares represent upregulation. The scale bar is shown as the log_2_ (fold change) value

We also identified several TFs that were differentially expressed in response to ABA and NDGA treatments (Fig. 11, Additional file 7). Exogenous ABA upregulated the expression of *3 APETALA2/ethylene-responsive elements* (*AP2/ERF*, Zj.jz042635005, Zj.jz044705012, and Zj.jz044537132), two *zinc finger proteins* (Zj.jz028519044 and Zj.jz006119044), 1 *NAC domain-containing protein* (Zj.jz007373078), and 1 *WRKY* gene (Zj.jz015029046). In addition, ABA downregulated the expression of 2 *AP2/ERF*s (Zj.jz044531026 and Zj.jz034557065), 2 *bHLH*s (Zj.jz042793020 and Zj.jz042921104), 2 *bZIP*s (Zj.jz0137047 and Zj.jz037039133), 1 *zinc finger protein* (Zj.jz041823042), 3 *homeobox* (*HB*) transcription factors (Zj.jz029235034, Zj.jz039989051, and Zj.jz043799010), 1 *heat-shock* TF (*HSF*, Zj.jz004979138), 6 *MYB*s (Zj.jz004979253, Zj.jz005919060, Zj.jz008869110, Zj.jz019661008, Zj.jz041121020, Zj.jz043819011, and Zj.jz044713001), and 2 *WRKY*s (Zj.jz034949026 and Zj.jz042571063).

The NDGA also induced the expression of several DEGs related to TFs, including 2 *AP2/ERF*s (*ZjERF52*, Zj.jz034557065 and Zj.jz001627224), 1 *bHLH* (Zj.jz031003007), 1 *zinc figure protein* (Zj.jz017079223), and 1 *MYB* (Zj.jz044713001). In addition, the downregulated genes included 2 *bHLH*s (Zj.jz015029086 and Zj.jz016733025), 5 *zinc finger proteins* (Zj.jz038707050, Zj.jz042849020, Zj.jz038181028, Zj.jz037267016, and Zj.jz035687008), 1 *HB* (Zj.jz006473038), and 2 *MYB*s (Zj.jz022481098 and Zj.jz014311034).

### Validation of transcriptomic expression levels

Seventeen genes were selected to validate the transcriptomic expression levels by qPCR. The correlation coefficient between the RNA-seq data and relative expression ranged from 0.838–1.0 (Additional file 8), thereby confirming the reliability of the RNA-seq data.

## Discussion

### Dynamic accumulation of endogenous ABA associated with Chinese jujube fruit ripening

The dynamic accumulation of endogenous ABA during jujube fruit development and ripening was investigated in our study. Several studies have suggested that endogenous ABA is synthesized from pulp tissues and not transported from seeds [8, 11, 23]. The initial relatively high content of endogenous ABA at the YF stage in jujube could induce the uptake of assimilation products and the accumulation of sucrose [8, 9], while the rapid increase at the WM stage in response to the onset of ripening correlated with pigment accumulation, sugar metabolism, and cell wall degradation in the fruit [24]. The increase in ABA has also been reported in both climacteric and non-climacteric fruit, such as those of tomato [5], peach [25], persimmon [26], grape [24], strawberry [27], and watermelon [11]. ABA is important in inducing the onset of jujube fruit ripening because of the limited emission of ethylene at the WM stage of ‘Dongzao’ fruit reported in our last study [7]. Ethylene production slightly increased in the FR fruit, and ABA decreased simultaneously. We proposed that a complementary mechanism may exist, as *SlNCED1 RNAi* tomato plants contained lower contents of ABA but presented a higher production of ethylene [28]. Nevertheless, these findings suggest a role for ABA in regulating the onset of jujube fruit ripening and maintaining ripening processes.

The expression of ABA biosynthesis and metabolic pathway genes during jujube fruit development and ripening suggested a balancing role for regulating the expression of *NCED*, *CYP707A*, and *BG* genes and determining the accumulation of endogenous ABA [5, 29, 30]. Although the *ZjNCED3* expression level was not high, it tended to increase, which correlated with ABA accumulation; the *ZjNCED3* protein also showed an amino acid sequence similarity of 64% with the *AtNCED6* (AT3G24220) protein, which was crucial in controlling ABA biosynthesis in *Arabidopsis*. These results indicated *ZjNCED3* could also be important in determining ABA biosynthesis in Chinese jujube fruit. Although the expression of *ZjNCED1* and *ZjNCED2* failed to parallel the ABA accumulation, these genes may also participate in this process; for example, *CsNCED2* appeared to play a subsidiary role in ABA accumulation, as the gene product was restricted to chromoplast-containing tissue in *Citrus* fruit [31]. While *CYP707A*s were shown to catalyze ABA degradation [32, 33], we found that the expression of *ZjCYP707A3*, *ZjCYP707A4*, and *ZjCYP707A5* decreased at the onset of jujube fruit ripening, suggesting a developmental stage-dependent expression pattern [27], which was also reported in watermelon (*Cla020637* and *Cla016011*) [11] and mulberry (*MaCYP707–1*-*4*) [34]. In addition, *ZjCYP707A2* showed increased expression at the FR stage, which we propose is related to the decrease in ABA content at that stage. With respect to *BG*s, the encoded enzymes catalyze the hydrolysis of glycosylated-ABA and rapidly increase the content of free ABA in plant tissues [35]. Our results suggested that *ZjBG4*, *ZjBG5*, *ZjBG8*, and *ZjBG9* are potentially involved in regulating ABA accumulation due to their expression patterns being consistent with dynamic changes in ABA [5, 23]; inhibiting the expression of *FaBG3* decreased the ABA content and delayed strawberry fruit ripening [36]. In cucumber, two *BG*s were also identified as being involved in the regulation of ABA accumulation [23].

The expression of several other genes involved in the ABA biosynthesis pathway was also determined in our study, although there are few reports related to their expression during fruit ripening. *ZjZEP* was consistently expressed during jujube fruit development and ripening, and its expression was also not related to ABA accumulation in *Vaccinium myrtillus* fruit [37]. *ZjABA2* showed increased expression following dynamic changes in ABA, while *AtABA2*-overexpressing plants also presented relatively high ABA accumulation [38]; however, correlations were not detected during fruit ripening of *Vaccinium myrtillus* [37]. In jujube, only *ZjAAO3* showed an expression consistent with dynamic changes in ABA; *AAO*s belong to a small gene family whose members have been purported to be functionally redundant, and only *AtAAO3* participates in ABA biosynthesis in *Arabidopsis* [39, 40]. In addition, the expression of *ZjABA3–1* conformed to that of *ZjAAO3*; *ABA3* encodes a molybdenum cofactor sulfur enzyme and functions together with *AAO3* [30]. A previous study also showed that expression of *AtABA3* was positively correlated with the ABA content in *Arabidopsis* [41].

### Transcription of ABA signaling pathway genes during Chinese jujube fruit development and ripening

The ABA signaling pathway, consisting of *PYR*/*PYL*/*RCAR*-*PP2C*-*SnRK2,* has been widely reported in plants [5]. In our study, *ZjPYR1* and *ZjPYL5* were expressed at a relatively high level in immature fruit, but their expression was low after the onset of ripening, while *ZjPYL8* and *ZjPYL9* showed the opposite trend, with relatively high expression during ripening. According to the results of a phylogenetic analysis (Additional file 9), *ZjPYR1* belonged to clade III, and *ZjPYL5* belonged to clade II. These two clades also showed a negative correlation between their expression and ABA accumulation in *Citrus* fruit (*CsPYR1*, *CsPYL4*, and *CsPYL5*) [42]. *ZjPYL8* and *ZjPYL9* belonged to clade I, and the genes involved in this clade were found to have positively accumulated along with the increase in ABA, as did the *CsPYL8* and *CsPYL9* genes in *Citrus* [42]; *SlPYL1*, *SlPYL2*, and *SlPYL3* in tomato [43]; and *FaPYR1* in strawberry [44]. Therefore, our findings suggest that *ZjPYR1* and *ZjPYL5* are key genes for ABA signaling in immature jujube fruit, while *ZjPYL8* and *ZjPYL9* are more important for maintaining signaling during fruit ripening processes.

PP2C proteins act as negative regulators in ABA signaling [45], and they can combine with receptors and form into components of protein kinase complexes, which regulate fruit sensitivity to ABA signaling [42, 46, 47]. Our study identified eight genes belonging to the *PP2C*-A subfamily, and five of them (*ZjPP2C4*–*8*) were upregulated along with ABA accumulation. Several previous studies also showed that the expression of partial *PP2C*s accumulated during fruit ripening in *Citrus* (*CsABI1*, *CsHAB1*, *CsAHG1*, *CsAHG3*, and *CsHAI3*), tomato (*SlPP2C1* and *SlPP2C5*), and grape (*VvPP2C3*, *VvPP2C6*, *VvPP2C7*, and *VvPP2C9*) [42, 43, 48]. However, more evidence is still needed to elucidate the relationships between *PP2C*s and sensitivity to ABA signaling.

Additionally, seven *SnRK2*s were identified in our study and were classified into three clades according to studies in both *Citrus* and tomato [42, 43]. However, only clade III was suggested to be involved in the ABA signaling pathway due to the enriched Asp (D) residues, which were located within the C-terminal sequences and were known as kinase binding sites [49]. Our study found that only *ZjSnRK2–2* and *ZjSnRK2–5* belonged to this clade (Additional file 9), and they did not show any significant correlations between their transcripts and ABA content. This phenomenon was consistent with previous findings in *Citrus*, tomato, and apple fruit [42, 43, 50]. For clades I and II, *ZjSnRK2–3*, *ZjSnRK2–4*, and *ZjSnRK2–7* also accumulated with the increase in ABA content, which suggests that these genes participate in ripening regulation but are not involved in ABA signaling [51]. Nevertheless, *ZjSnRK2–2* and *ZjSnRK2–5* are relatively important for maintaining ABA signaling in jujube fruit.

### ABA acts as a positive regulator in Chinese jujube fruit ripening

To further investigate the role of ABA in modulating jujube fruit ripening, WM ‘Dongzao’ fruit were treated with a low concentration of ABA or NDGA. Our results showed that ABA treatment promoted a rapid accumulation of endogenous ABA and partly increased fruit respiration and ethylene production. These results were similar to those of a recent study showing that 50 mg L^− 1^ ABA promoted WM ‘Dongzao’ fruit ripening and reduced fruit quality during postharvest storage [52]. In addition, our research showed that 50 mg L^− 1^ NDGA inhibited the increase in endogenous ABA at 3 DAT, significantly decreased fruit respiration, but slightly affected ethylene production in our study. NDGA is an inhibitor of ABA biosynthesis because it could interact with NCED enzymes with regard to its permeation speed [53]. Therefore, NDGA reduced ABA biosynthesis but did not completely suppress the process [54, 55]. Thus, these results suggested that ABA acted as a potential positive regulator in jujube fruit ripening.

The comparative transcriptome analysis provided an overview of DEGs correlated with altered metabolic processes in response to the treatments. Previous studies have suggested that the transition underlying fruit ripening induced by ABA is associated with the suppression of vegetative growth metabolism and the activation of ripening-specific pathways [14, 56]. In our study, genes related to photosynthetic metabolism and several secondary metabolites were found to be somewhat downregulated by ABA (Additional file 5). Photosynthetic metabolism is regarded as a switch that induces the transition to fruit ripening via downregulated gene expression in response to exogenous ABA [13, 14, 57]. Moreover, genes involved in the metabolism of flavones, flavanols and flavonoids were downregulated by ABA at 1 DAT, suggesting a possible stress response of jujube fruit to exogenous ABA. In grape, the expression of genes associated with phenylpropanoid biosynthesis and flavonoid biosynthesis was also downregulated by ABA at 20 h posttreatment, but the expression was upregulated at 44 h [14]. We speculated that secondary metabolism would be positively induced by ABA after 1 DAT because of its positive role in regulating secondary metabolites; this process is common to tomato, grape and strawberry [13, 16, 58].

On the other hand, we found that genes involved in lipids, cell wall modification, starch, and sucrose metabolism were somewhat positively induced by ABA and NDGA, suggesting a correlation between ABA and ripening-related metabolism. In lipid pathways, altered gene expression participates in the modification of the cell membrane, the biosynthesis of aromatic compounds, and lignification induced by wounding [12, 19, 59, 60]. With respect to cell wall modification, genes related to cellulose synthesis, cell wall proteins, and cell wall degradation and modification were somewhat upregulated by ABA, indicating that ABA has a potential role in regulating changes in fruit texture [5, 17, 61]. In the starch biosynthesis pathway, genes encoding ADP-glucose pyrophosphorylase and isoamylase were downregulated by ABA, while NDGA downregulated the expression of genes controlling starch degradation, including two alpha-amylase- and a beta-amylase-encoding genes. These results suggested that ABA was involved in starch metabolism, just as *ZmEREB156* positively modulated starch biosynthesis via the synergistic effect of sucrose and ABA in maize [62]. In sucrose metabolism, genes encoding the vacuolar invertase, hexokinase, and sugar transporters were significantly induced by ABA, indicating that ABA promoted sucrose degradation but maintained the content of glucose and fructose, while the expression of these genes was inhibited after NDGA treatment. These results suggest a putative role of ABA in regulating sucrose metabolism, and genes related to sucrose metabolism were also found to be activated after 44 h in grape fruit treated with exogenous ABA [14]. Overall, our study provided comprehensive insight into the role of ABA in regulating jujube fruit ripening-related metabolism, and additional studies are still needed to elucidate the complex regulatory mechanism involved.

### The regulatory network of ABA in modulating Chinese jujube fruit ripening

**Fig. 12:**
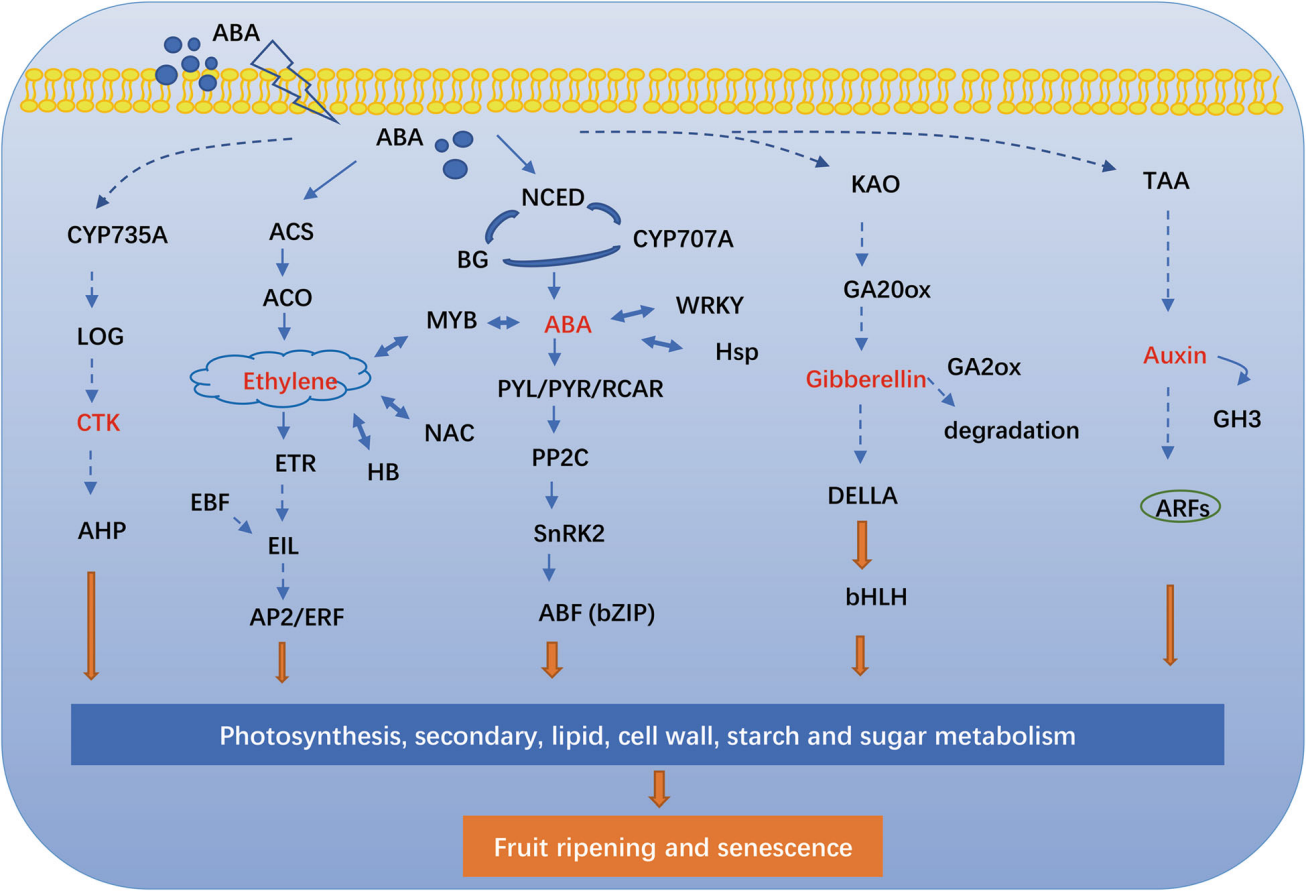
Putative regulatory network for Chinese jujube fruit ripening

The crosstalk between ABA and other plant hormones and TFs was analyzed in our study via transcriptomic data (Fig. 12). Exogenous ABA promoted a rapid accumulation of endogenous ABA in treated fruit but inhibited the expression of *ZjNCED2*, indicating a negative feedback regulation in response to exogenous ABA, as was the case for *CsNCED1* in *Citrus* and *LeNCED2* in tomato [20, 54]. *ZjBG4* and *ZjBG5* were upregulated in response to ABA, and these two genes showed a positive response to ABA accumulation during fruit ripening, which is crucial in regulating the ABA content [11, 23]. Conversely, NDGA treatment promoted the expression of *ZjCYP707A1* but inhibited *ZjBG5*, thereby inhibiting the increase in ABA content at 3 DAT. These results corroborated the role of transcriptional balance among *NCED*, *CYP707A*, and *BG* in maintaining ABA accumulations [11, 19].

We also found that genes involved in ethylene biosynthesis and perception, including *ZjACS7*, *ZjACO3*, and *ZjERS2*, were upregulated by ABA; similar results were also found in tomato (*LeACS2*, *LeACS4*, and *LeACO1*) and grape (*VvACO1*), suggesting that ABA may act as an upstream regulator of the ethylene pathway [8, 25, 55]. With respect to the ethylene signaling pathway, *ZjERS2* was upregulated by ABA. The increased expression responds to the degradation of receptors induced by the binding with ethylene [63, 64]. The genes involved in the signaling pathway were rarely induced by the treatments, although the expression of the *ZjEBF2* gene was upregulated by ABA. Moreover, *EBF* regulates the expression of *ethylene-insensitive 3-like* (*EIL*) at the posttranscriptional level [65, 66]; therefore, this result also indicated an indirect regulation of the ethylene signaling pathway by ABA.

In our study, the expression of several genes involved in the biosynthesis, metabolism, and signaling of GA, auxin, and cytokinin; *GA20ox*, *CYP735A*, and *LOG*, which are involved in the biosynthesis of GA and cytokinin; a *DELLA* protein involved in GA signaling, and an *AHP* gene involved in cytokinin signaling was downregulated in response to exogenous ABA. ABA also upregulated the expression of *GA2ox,* which regulates the degradation of GA. In addition, NDGA inhibited the expression of genes (*TAA* and *GH3*) involved in auxin biosynthesis. There is evidence suggesting that GA, auxin, and cytokinin decrease during fruit ripening in strawberry, grape, and tomato [67–69]. Moreover, litchi, strawberry, and sweet cherry fruit treated with these phytohormones inhibit or delay fruit ripening processes [15, 19, 22, 70]. Therefore, ABA would crosstalk with hormones which negatively correlated with ripening.

With respect to the TFs induced by ABA and NDGA, DEGs related to the *AP2/ERF*, *NAC*, *WRKY*, *bHLH*, *bZIP*, *HB*, *MYB*, *zinc finger protein*, and *Hsf* TFs were identified. These TFs are extensively involved in fruit metabolism such as phytohormone metabolism and signaling, the accumulation of flavonoids and anthocyanins, and the response to stress [6, 71]. Exogenous ABA inhibited the expression of *ZjERF34* and *ZjERF52*, which were previously suggested to be downregulated during ripening in our last study, while *ZjDREB1* was upregulated by ABA and was also found to be positively correlated with ripening [72]. In addition, two *bZIP*s were downregulated by ABA, and Zj.jz037039133 is probably located at the end of ABA signaling as an ABA response factor; this factor was suggested to be involved in the regulation of ABA and phenol accumulation in grape [73]. Our study also identified 6 MYB TFs that were downregulated by ABA and a TF that has an extensive role in modulating fruit coloration and texture [74]. A number of *zinc finger proteins* were found to be induced by exogenous treatment, and C2HC-type *zinc finger proteins* could respond to the resistance of oxidative stress induced by exogenous ABA [75]. We also found that the *NAC* gene was upregulated by ABA and was found to be involved in the regulation of ethylene signaling, fruit pigmentation and fruit softening [76]. Overall, the identification of these TFs provided us with broad insight into the complex regulation of ABA involvement in modulating jujube fruit ripening, and the potential mechanism of these TFs should be preferentially studied in the future.

## Conclusions

Our study provided comprehensive insights into the role of ABA in Chinese jujube fruit ripening and revealed a positive correlation between the onset of fruit ripening and the accumulation of endogenous ABA, which was modulated by the interaction among the expression of *NCED* (*ZjNCED3*), *CYP707A* (*ZjCYP707A2*), and *BG*s (*ZjBG4*, *ZjBG5*, *ZjBG8*, and *ZjBG9*). The signaling pathway consisting of a receptor (*ZjPYL8*), *PP2C*s (*ZjPP2C4*, *ZjPP2C5*, *ZjPP2C6*, *ZjPP2C7*, and *ZjPP2C8*) and *SnRK2*s (*ZjSnRK2–2* and *ZjSnRK2–5*) was important in regulating fruit sensitivity to ABA. In addition, ABA had a dominant role in modulating jujube fruit ripening, and our study further elucidated ABA-associated metabolism and predicted a network of jujube fruit ripening regulations. These findings would be helpful for further improvements to strategies for jujube fruit storage and for gaining insights into understanding complex non-climacteric fruit ripening.

## Methods

### Plant materials, treatments and storage

Fruits of a popular *Z. jujuba* cultivar, ‘Dongzao’, were harvested from adult trees grown in our experimental orchard in Xuzhuang, Dali, Shaanxi, China (N34.52, E109.56). The sampling periods were selected according to days after full blooming (DAB) and changes in peel color: 15 DAB, young fruit (YF); 30 DAB, enlarging fruit (E1); 40 DAB, E2; 55 DAB, E3; 70 DAB, E4; 85 DAB, white mature (WM); 100 DAB, beginning red (BR); 110 DAB, half-red (HR); and 115 DAB, full-red fruit (FR). Three biological replicates of fifteen fruits were collected, and their weights were measured at each sampling period. The fruits were cut into small cubes, immediately frozen in liquid nitrogen, and stored at − 80 °C for subsequent experiments.

The fleshy fruits of ‘Dongzao’ in the WM stage (onset of ripening) were hand harvested from five trees per treatment. The fruits were kept at 15 °C for 6 h until they were transferred to our lab, after which they were cleaned in water and then air dried for 30 min at room temperature (20 °C). Then, the fruits were randomly divided into three groups and immersed in a solution of 50 mg L^− 1^ (±)-ABA (Sigma-Aldrich), 50 mg L^− 1^ NDGA (Sigma-Aldrich) or distilled water (control) for 10 min. After treatment, the fruits of each group were placed in a 10 L uncovered plastic container and stored in darkness at 20 °C. Fruits on the 1st, 3rd, 5th, 7th and 9th days after treatment (DAT) were cut into pieces, frozen in liquid nitrogen, and stored at − 80 °C.

### Extraction and determination of endogenous ABA

Endogenous ABA was extracted twice at 4 °C for 12 h from 1.0 g of crushed fruit with 4 mL of 80% methanol containing 200 mg L^− 1^ butylated hydroxytoluene and 500 mg L^− 1^ citric acid monohydrate [77]. After centrifugation, the supernatants were combined and dried under nitrogen at 35 °C_._ The residues were dissolved in 0.8 mL of 80% methanol and were filtered through a 0.22 μm filter membrane.

Quantification of ABA level was modified from a previously published method [78] and performed using high-performance liquid chromatography (LC-20AT, Shimadzu, Kyoto, Japan) coupled with electrospray tandem mass spectrometry (QTRAP 5500, AB SCIEX, USA). In detail, separation of ABA was achieved using a C18 column (2.1 × 150 mm, 5 μm, Eclipse XDB, Agilent, USA) at 40 °C with the following solvent gradient (solvent A, methanol; solvent B, 0.05% formic acid): 0–8 min, 30% B; 8–15 min, linear increase to 100% B; 15–17 min, 100% B; and 17–22 min, linear decrease to 30% B, with a flow rate of 0.50 mL s^− 1^ and an injection volume of 10.0 μL. For mass spectrometric analyses, the following conditions were used: IS, Turbo Spray (−), − 4000; FP, − 400; EP, − 10; CXP, − 4; CUR, 10; CAD, 5; TEM, 450; GS1, 50; GS2, 55; and ihe, ON. The retention time and mass spectrometry information concerning ABA were determined with a standard substance ((±) ABA, Sigma-Aldrich), and the following conditions were ultimately selected: Q1, 263.3; Q3, 153.2; DP, − 24; and CE, − 16. External calibration curves were prepared using different concentrations of standard ABA dilution. Three independent biological replicates were prepared for each sample.

### Measurement of respiration rate and ethylene production during fruit storage

The respiration rate was determined every day during storage at 14:00 using an LI-6400XT Photosynthesis System (Lincoln, NE, USA), which was attached to a 0.45 L cylindrical chamber with an air flow rate of 700 μmol s^− 1^. The stable variation in ΔCO_2_ of approximately 100 g of fruit was used to calculate the respiration rate [79]. Three independent biological replicates were measured for each sample.

Extraction and determination of ethylene production were performed according to our previous published method [7]. Ethylene was collected by sealing approximately 100 g of fruit in a 0.5 L glass bottle for 2 h at room temperature and drawing 5 mL of headspace gas into a 20 mL penicillin bottle filled with water [7]. Gas samples were collected every day during storage and determined together after collection. One milliliter of gas from the penicillin bottle was injected into a gas chromatograph (Trace GC ULTRA2010, Thermo, USA) fitted with a flame ionization detector and a Porapak 80–100 packed column (200 × 3 mm). The oven, injector, and detector temperatures were 70, 70, and 150 °C, respectively. The carrier gas (N_2_, H_2_, and air) flow rates were 35, 35, and 350 ml min^− 1^, respectively. Three independent biological replicates were measured for each sample.

### Identification of genes involved in ABA biosynthesis, metabolism, and the signaling pathway

Based on the jujube genome dataset (10.5061/dryad.83fr7) [80], the ABA biosynthesis, metabolism, and signaling pathway genes were identified and confirmed by BLASTN and SMART analyses [81]. The sequence information of these genes is listed in Additional file 10. The predicted proteins were aligned with the known protein sequences of grape, tomato, and *Arabidopsis*, and their sequences were subjected to KEGG analysis (https://www.genome.jp/kegg/pathway.html). Phylogenetic trees for *NCED*, *CYP707A*, *BG*, *PYR/PYL/RCAR*, *PP2C*, and *SnRK2* genes were generated using the neighbor-joining method and 1000 bootstrap replications by MEGA 7.0 [82].

### RNA isolation, cDNA library construction and Illumina sequencing

The control and ABA- and NDGA-treated fruits at 1 DAT (two biological replicates) were subjected to transcriptome sequencing. Total RNA was isolated using a plant RNA extraction kit (TaKaRa, Dalian, China) and digested with RNase-free DNase I. Thereafter, the amount of RNA was measured by spectrophotometric analysis, and its quality was further verified by agarose gel electrophoresis. A total of 3 μg of RNA per sample was used for cDNA library preparation with a NEBNext Ultra RNA Library Prep Kit (NEB, USA), and the resulting PCR products were purified and quantified on an Agilent Bioanalyzer 2100 system. Sample labeling was performed on a cBot Cluster Generation System using a TruSeq PE Cluster Kit v3-cBot-HS. Afterwards, the libraries were sequenced on an Illumina HiSeq PE150 platform. The raw sequencing data were subsequently submitted to the Sequence Read Archive (https://www.ncbi.nlm.nih.gov//sra/) (Additional file 11).

### Bioinformatic processing of transcriptome sequencing data

Raw sequencing data generated from the Illumina HiSeq X platform were processed to remove low-quality reads containing adapters and a high rate of poly-N (> 10%) using an in-house Perl script. Simultaneously, the values of the Q20, Q30, and GC content were calculated for the clean data. Afterwards, the clean data were aligned with the reference jujube genome sequences using TopHat v2.0.9 [83] and were further processed for gene expression level quantification by HTSeq v0.6.1 [84]. The relative gene expression abundance was estimated with the value of reads per kilobase of exon model per million mapped reads (RPKM) from the mean of two biological replicates [85]. For identification of differentially expressed genes (DEGs), an edgeR program [86] in OmicShare tools, a free online platform for data analysis (http://www.omicshare.com/tools), was used with a fold change (FC) threshold ≥2 and an false discovery rate (FDR) ≤ 0.05. The use of edgeR allowed comparative analysis within two replicates, and it had been used in several previous papers [12, 87, 88]. The functional enrichment of DEGs was determined using Gene Ontology (GO) and pathway analysis tools within the OmicShare platform [89]. We also used MapMan 3.6.0RC1 software to enrich the putative functional annotation of the DEGs [90, 91].

### Quantitative real-time PCR validation for transcriptome expression levels

Total RNA was extracted using a plant RNA extraction kit (TaKaRa), and 200 ng of high-quality RNA was subsequently prepared for first-strand cDNA synthesis using a PrimeScript RT reagent kit with gDNA Eraser (TaKaRa). qPCR was then performed using a SYBR Premix Ex Taq II kit (TaKaRa) with a total volume of 10 μL, which comprised 1.0 μL of cDNA, 5.0 μL of SYBR premix solution, 0.4 μL of forward/reverse primers and 3.2 μL of dH_2_O. The thermal program for qPCR in a Roche LightCycler 96 system was set using the following conditions: 95 °C for 30 s; 40 cycles of amplification of 5 s at 95 °C, 30 s at 58 °C, and 30 s at 72 °C; and a default dissociation stage. The relative expression of each gene was normalized to that of a reference gene, *ZjUBQ* (Zhang et al. 2015), and was ultimately calculated using the 2^-△Ct^ method (Livak and Schmittgen 2001). Sequences of the primers used for qPCR are listed in Additional file 10.

### Statistical analysis

Statistical analysis was performed using the Duncan multiple range test (MRT) at the *p* < 0.05 level in SPSS 19.0. The error bars in the figures represent the standard deviations of three biological replicates.

## Additional files

Additional file 1: Statistic information of RNA-seq data. (XLSX 11 kb)

Additional file 2: Statistic information of mapping data. (XLSX 11 kb)

Additional file 3: GO enrichment for DEGs at DAT1. (XLSX 17 kb)

Additional file 4: KEGG pathway enrichment for DEGs (Level3) at DAT1. (XLSX 27 kb)

Additional file 5: DEGs related to metabolism pathways by MapMan. (XLSX 68 kb)

Additional file 6: DEGs related to hormone metabolism and signaling. (XLSX 21 kb)

Additional file 7: DEGs related to transcription factors involved in ripening regulation. (XLSX 27 kb)

Additional file 8: RT-qPCR validation of digital expression patterns revealed by RNA sequencing. A number of 17 genes were selected to validate the transcriptomic expressions by qPCR. The correlation coefficient between the RNA-seq data and relative expression ranged from 0.838–1.0, thereby confirming the reliability of the RNA data. (DOCX 241 kb)

Additional file 9: Phylogenetic analyses for NCED, CYP707A, BG, PYR/PYL/RCAR, PP2C, and SnRK2 genes. The trees were generated by the multiple alignments with putative proteins from Arabidopsis, grape, and tomato which were uploaded in the KEGG database using MEGA 7.0. The Bootstrap value was set into 1000 (Kumar et al. 2016). (DOCX 983 kb)

Additional file 10: Sequences for ABA pathway genes. (XLSX 124 kb)

Additional file 11: Accession of clean reads submitted to sequence read archives (SRA) of NCBI. (XLSX 10 kb)

## Abbreviations

AAO: Abscisic aldehyde oxidase
ABA: Abscisic acid
ABA2: xanthoxin dehydrogenase
ABA3: molybdenum cofactor sulfurtransferase
ACO: 1-aminocyclopropane-1-carboxylate oxidase
ACS: 1-aminocyclopropane-1-carboxylate synthase
AHP: histidine-containing phosphotransferase
AOG: ABA-glucosyltransferase
AP2/ERF: APETALA2/ethylene-responsive element
BG: Beta-glucosidase
BR: Beginning red
CYP707A: ABA-8′-hydraxylase
DAB: Day after blooming
DAT: Day after treatment
DEG: Differentially expressed gene
E: Enlarging fruit
EBF: Ethylene insensitive 3-binding F-box
EIN3/EIL: Ethylene insensitive 3-like
ERS: Ethylene response
ETR: Ethylene receptor
FC: Fold change
FDR: False discovery rate
FR: Full-red
GA: Gibberenllin
GA20ox: GA-20-oxidase
GA2ox: GA-2-oxidase
GH3: Gretchen hagen 3
GO: Gene ontology
HB: Homeobox transcription factor
HR: Half-red
HSF: Heat-shock transcription factor
KAO: *ent*-kaurenoic acid hydroxylase
LOG: cytokinin riboside 5′-monophosphate phosphoribohydrolase
MRT: Multiple range test
NCED: Nine-cis-epoxycarotenoid dioxygenase
NDGA: Nordihydroguaiaretic acid
PP2C: Protein phosphatase 2C
PYR/PYL/RCAR: Pyrabatin resistance/pyrabatin resistance 1-like/regulatory component
SnRK2: Sucrose nonfermenting 1-related protein kinase 2
TAA: Tryptophan aminotransferase
TF: Transcription factor
WM: White mature
YF: Young fruit
ZEP: Zeaxanthin epoxidase
