# Supplementary material for: Transcript analyses reveal a comprehensive role of abscisic acid in modulating fruit ripening in Chinese jujube
Source: BMC Plant Biol. 2019 May 8;19:189. doi: 10.1186/s12870-019-1802-2 (PMC6505321; doi:10.1186/s12870-019-1802-2)

**Additional file 8.** RT-qPCR validation of digital expression patterns revealed by RNA sequencing. A number of 17 genes were selected to validate the transcriptomic expressions by qPCR. The correlation coefficient between the RNA-seq data and relative expression ranged from 0.838 -1.0, thereby confirming the reliability of the RNA data.


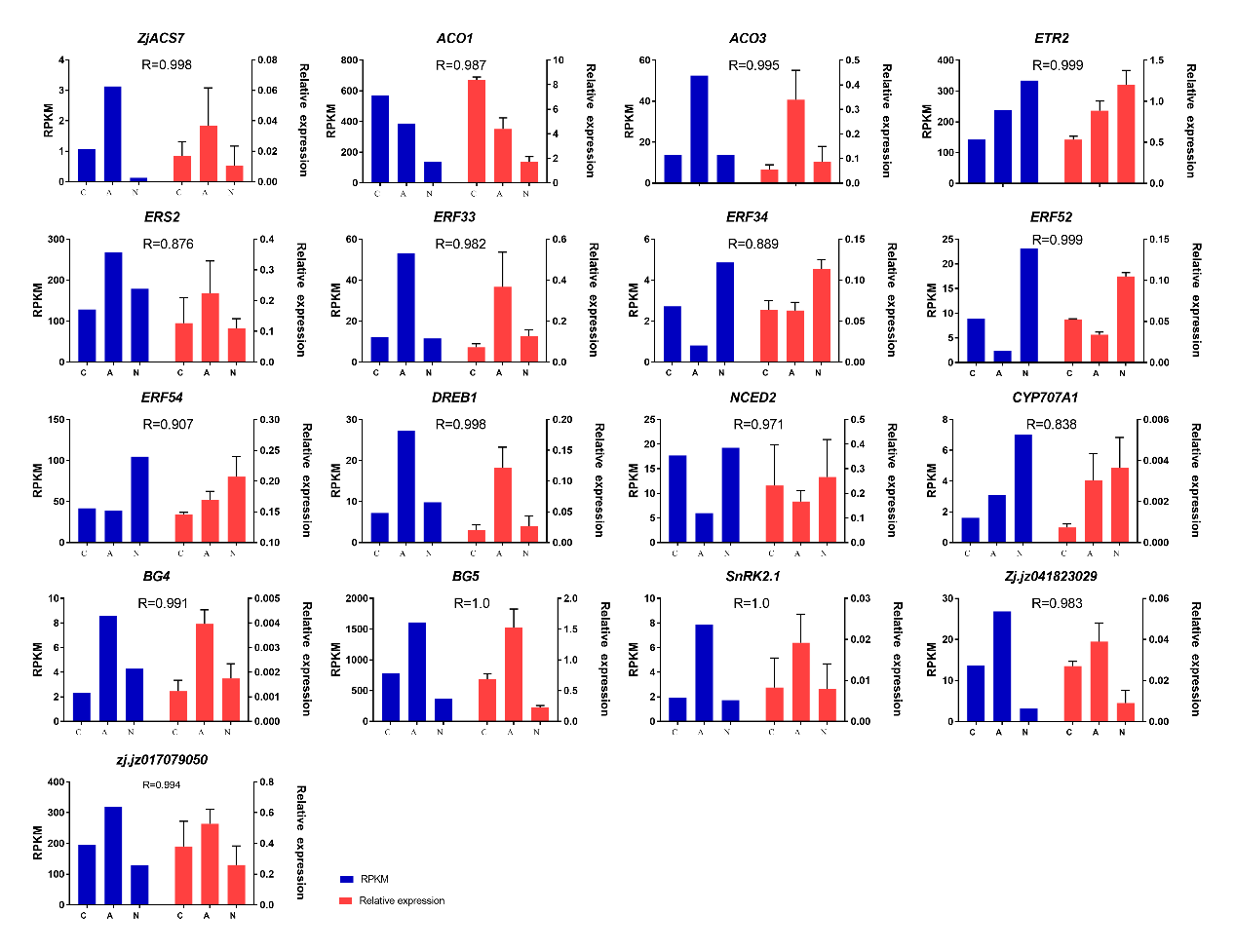

Supplement: Supplementary file 8 — RT-qPCR validation of digital expression patterns revealed by RNA sequencing. A number of 17 genes were selected to validate the transcriptomic expressions by qPCR. The correlation coefficient between the RNA-seq data and relative expression ranged from 0.838–1.0, thereby confirming the reliability of the RNA data. (DOCX 241 kb) [file 12870_2019_1802_MOESM8_ESM.docx]
