# Supplementary material for: Transcript analyses reveal a comprehensive role of abscisic acid in modulating fruit ripening in Chinese jujube
Source: BMC Plant Biol. 2019 May 8;19:189. doi: 10.1186/s12870-019-1802-2 (PMC6505321; doi:10.1186/s12870-019-1802-2)
Supplement: Supplementary file 9 — Phylogenetic analyses for NCED, CYP707A, BG, PYR/PYL/RCAR, PP2C, and SnRK2 genes. The trees were generated by the multiple alignments with putative proteins from Arabidopsis, grape, and tomato which were uploaded in the KEGG database using MEGA 7.0. The Bootstrap value was set into 1000 (Kumar et al. 2016). (DOCX 983 kb) [file 12870_2019_1802_MOESM9_ESM.docx]

**Additional file 9.** Phylogenetic analyses for *NCED*, *CYP707A*, *BG*, *PYR/PYL/RCAR*, *PP2C*, and *SnRK2* genes. The trees were generated by the multiple alignments with putative proteins from *Arabidopsis*, grape, and tomato which were uploaded in the KEGG database using MEGA 7.0. The Bootstrap value was set into 1000 (Kumar et al. 2016).

**(1) Phylogenetic trees for *NCED*, *CYP707A* and *BG* genes.**


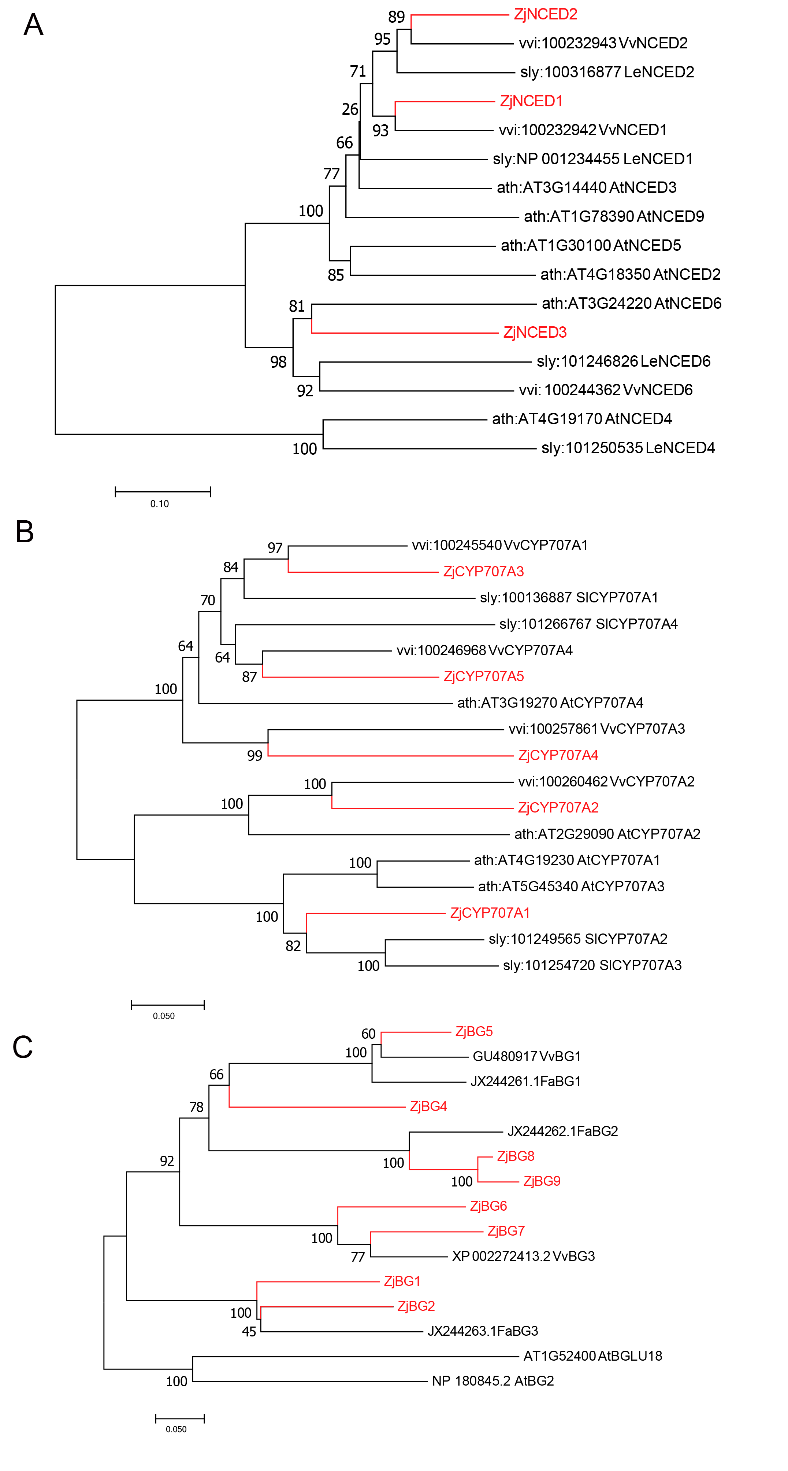


**(2) Phylogenetic trees for *PYR/PYL/RCAR* genes.**

The *PYR/PYL/RCAR* genes belongs to a small gene family. The trees were separated into three groups according to Romero et al. (2012). The group I included *ZjPYL8* and *ZjPYL9*, the group II contained *ZjPYL4* and *ZjPYL5*, and the group III include the *ZjPYL1* and *ZjPYL2*.


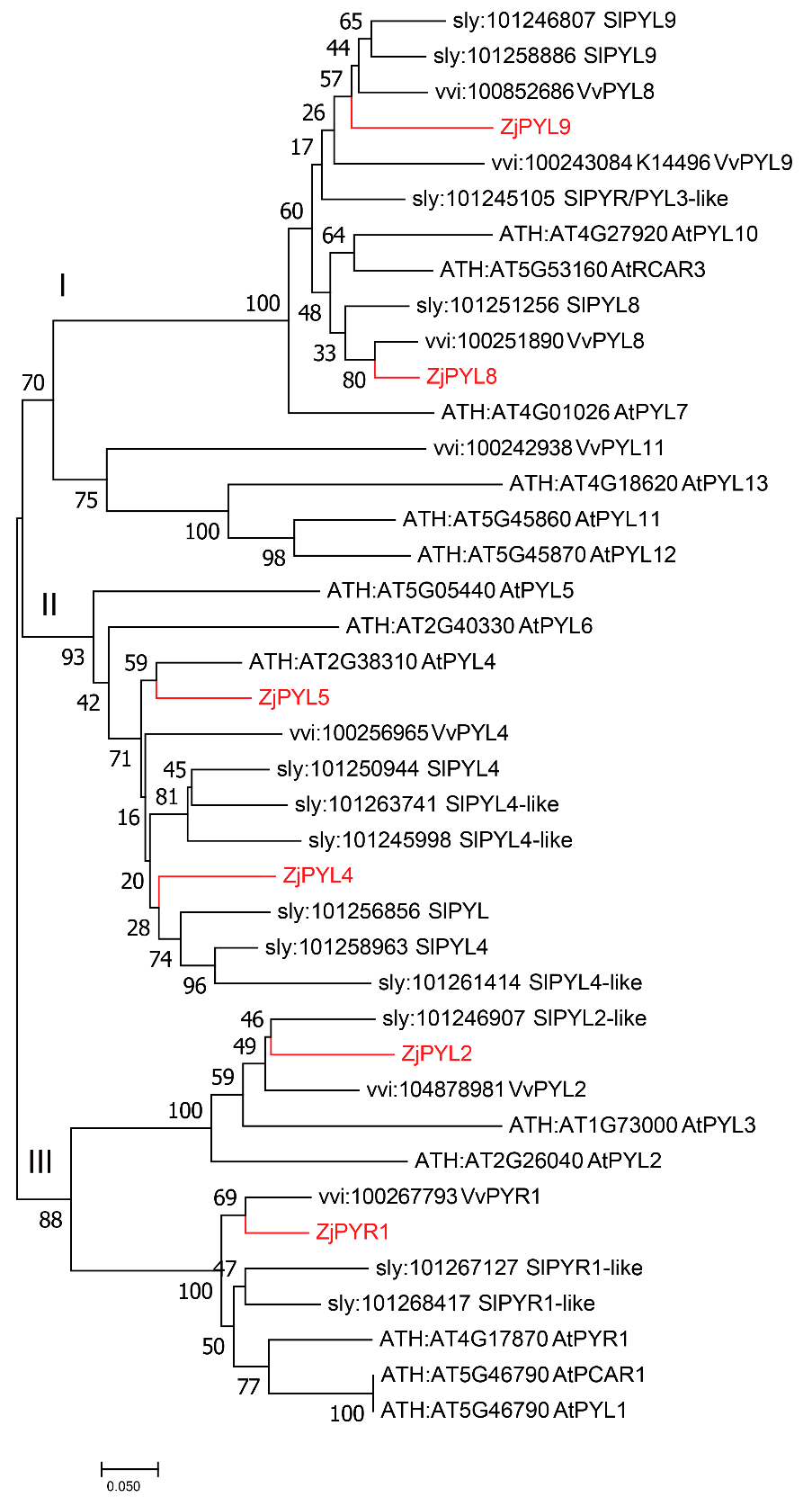


**(3) Phylogenetic trees for *PP2C* genes.**

The *PP2C* was a large gene family, and only the PP2C-A subfamily genes played a role in the ABA signaling pathway (Ben-Ari 2012). Thus, the PP2C-A subfamily genes were identified from the reference jujube genome. The 8 genes were divided into 3 groups. The group I included *ZjPP2C1*, *ZjPP2C7* and *ZjPP2C8*. The group II contained *ZjPP2C5* and *ZjPP2C6*. The groups III include *ZjPP2C2*, *ZjPP2C3*, and *ZjPP2C4*.


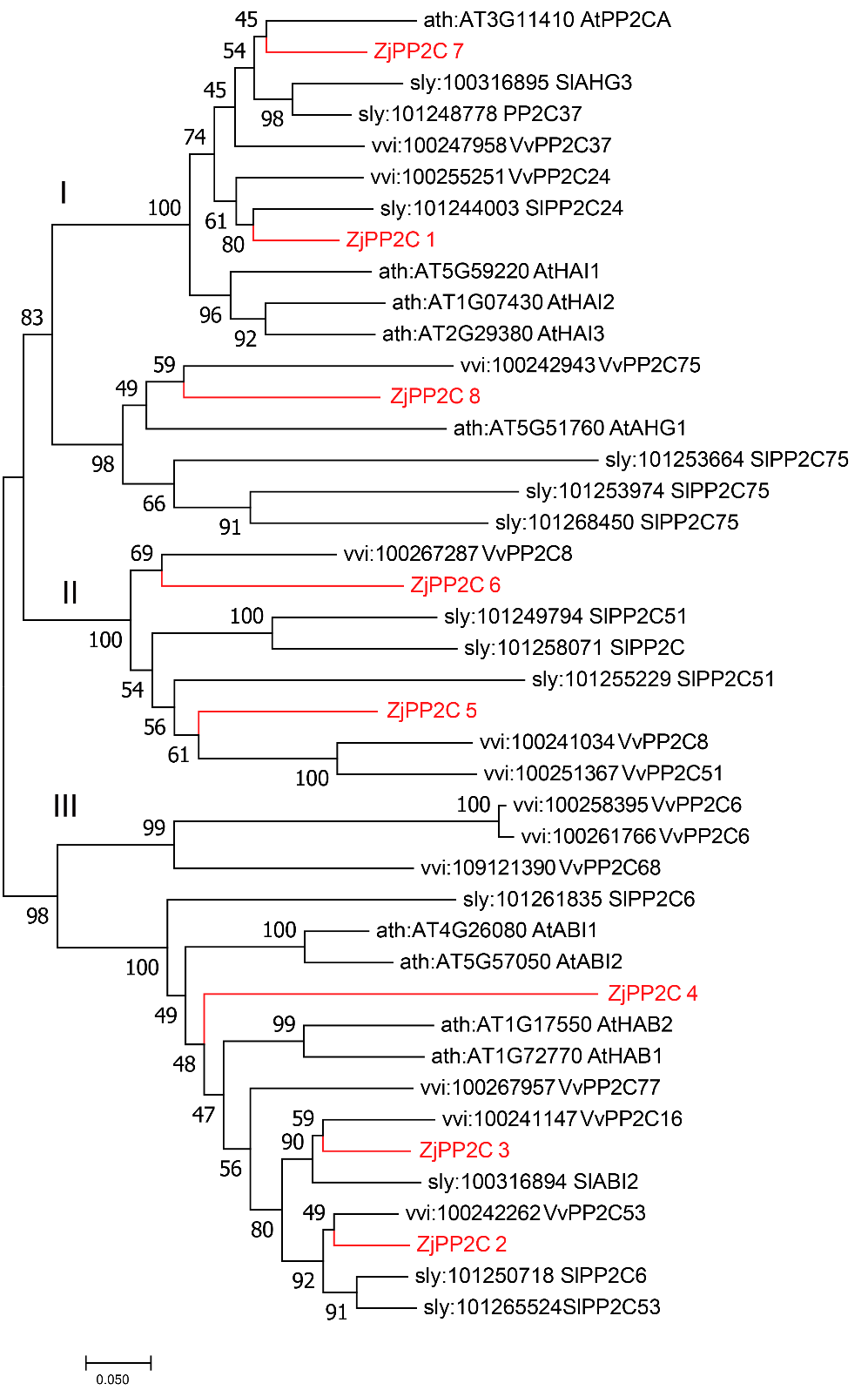


**(4) Phylogenetic trees for *SnRK2* genes.**

The *SnRK2* genes could be divided into 3 groups according to Romero et al. (2012). The group I included *ZjSnRK2-3* and *ZjSnRK2-4*, the group II included *ZjSnRK2-1*, *ZjSnRK2-6*, and *ZjSnRK2-7*, and the group III contained *ZjSnRK2-2* and *ZjSnRK2-5*. Previous studies suggested only genes involved in group III could be activated by ABA (Romero e al. 2012; Sun et al. 2011).


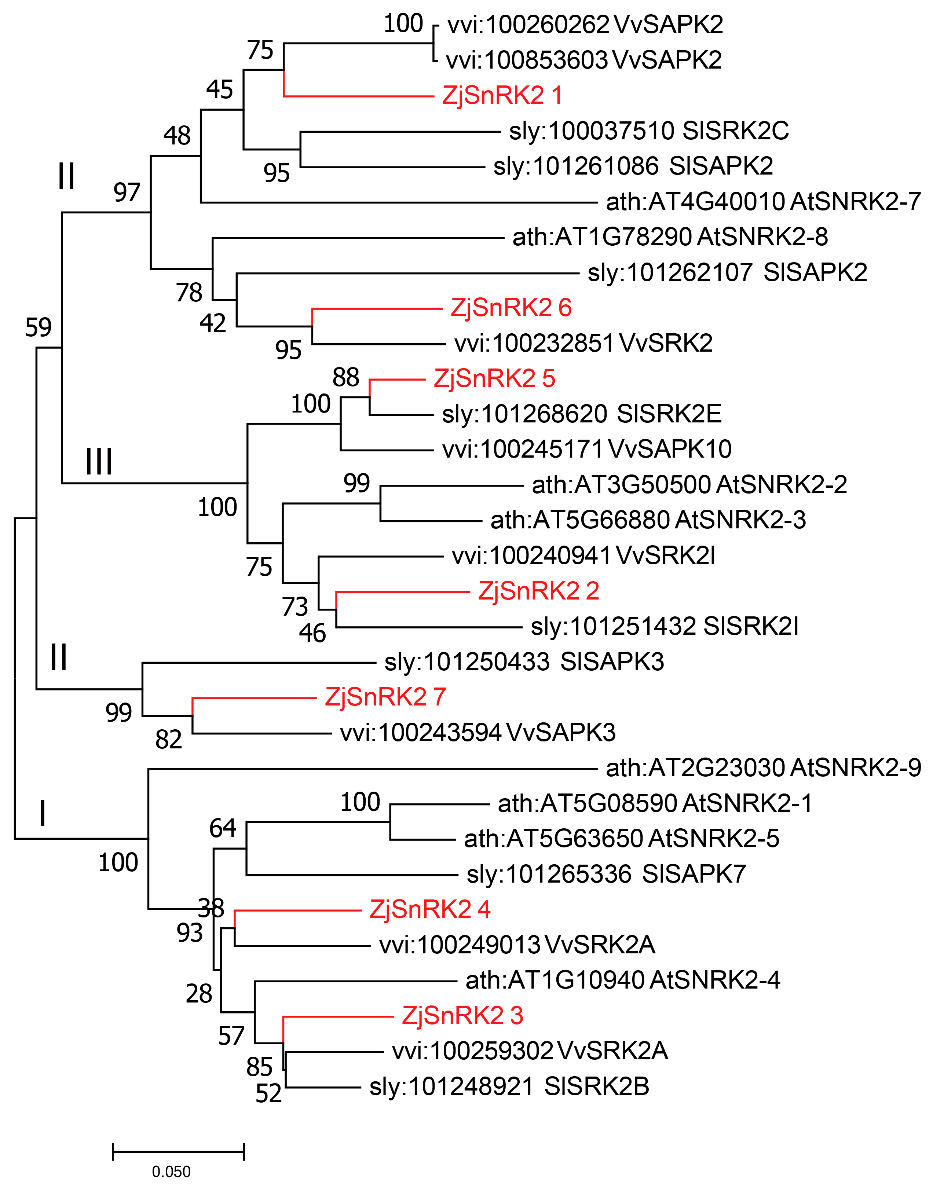


**Reference:**

Ben-Ari G. 2012. The ABA signal transduction mechanism in commercial crops: learning from *Arabidopsis*. Plant Cell Rep, 31(8):1357-1369

Kumar S, Stecher G, Tamura K. 2016. MEGA7: molecular evolutionary genetics analysis version 7.0 for bigger datasets. Mol Biol Evol, 33(7):1870-1874

Romero P, Lafuente M T, Rodrgo M J. 2012. The Citrus ABA signalosome: Identification and transcriptional regulation during sweet orange fruit ripening and leaf dehydration. J Exp Bot, 63(13):4931-4954

Sun L, Wang Y P, Chen P, Ren J, Ji K, Li Q, Li P, Dai S J, Leng P. 2011. Transcriptional regulation of *SlPYL*, *SlPP2C*, and *SlSnRK2* gene families encoding ABA signal core components during tomato fruit development and drought stress. J Exp Bot, 62(15):5659-5669
